# Exchange of medicinal plant information in California missions

**DOI:** 10.1186/s13002-020-00388-y

**Published:** 2020-06-15

**Authors:** Joe Rayl McBride, Rita Yolanda Cavero, Anna Liisa Cheshire, María Isabel Calvo, Deborah Lea McBride

**Affiliations:** 1 Department of Environmental Science, Policy and Management, University of California, Berkeley, CA USA; 2 Department of Environmental Biology, School of Sciences, University of Navarra, Irunlarrea 1, 31008 Pamplona, Spain; 3 School of Art, San Francisco State University, San Francisco, CA USA; 4 Department of Pharmaceutical Technology and Chemistry, School of Pharmacy and Nutrition, University of Navarra, Irunlarrea 1, 31008 Pamplona, Spain; 5 School of Nursing, Samuel Merritt University, Oakland, CA USA

**Keywords:** Medicinal plants, Native Americans, California Missions, Spanish priests, Information transfer, Californios

## Abstract

**Background:**

Missions were established in California in the eighteenth and nineteenth centuries to convert Native Americans to Christianity and enculturate them into a class of laborers for Californios (Spanish/Mexican settler). The concentration of large numbers of Native Americans at the Missions, along with the introduction of European diseases, led to serious disease problems. Medicinal supplies brought to California by the missionaries were limited in quantity. This situation resulted in an opportunity for the sharing of knowledge of medicinal plants between the Native Americans and the Mission priests. The purpose of this study is to examine the degree to which such sharing of knowledge took place and to understand factors that may have influenced the sharing of medicinal knowledge. The study also examines the sharing of medicinal knowledge between the Native Americans and the Californios following the demise of the California Missions.

**Methods:**

Two methods were employed in the study: (1) a comparison of lists of medicinal plants used by various groups (e.g., Native American, Mission priests, Californios) prior to, during, and after the Mission period and (2) a close reading of diaries, reports, and books written by first-hand observers and modern authorities to find accounts of and identify factors influencing the exchange of medicinal information.

**Results:**

A comparison of the lists of medicinal plants use by various groups indicated that only a small percentage of medicinal plants were shared by two or more groups. For example, none of the 265 taxa of species used by the Native Americans in pre-Mission times were imported into Spain for medicinal use and only 16 taxa were reported to have been used at the Missions. A larger sharing of information of medicinal plants took place in the post-Mission period when Native Americans were dispersed from the Missions and worked as laborers on the ranches of the Californios.

**Conclusions:**

Sharing of information concerning medicinal plants did occur during the Mission period, but the number of documented species was limited. A number of possible factors discouraged this exchange. These include (1) imbalance of power between the priests and the Native Americans, (2) suppression of indigenous knowledge and medical practices by the Mission priests, (3) language barriers, (4) reduction of availability of medicinal herbs around the Mission due to introduced agricultural practices, (5) desire to protect knowledge of medicinal herbs by Native American shaman, (6) administrative structure at the Missions which left little time for direct interaction between the priests and individual Native Americans, (7) loss of knowledge of herbal medicine by the Native Americans over time at the Missions, and (8) limited transportation opportunities for reciprocal the shipment of medicinal plants between California and Spain. Three possible factors were identified that contributed to a greater sharing of information between the Native Americans and the Californios in the post-Mission period. These were (1) more one-to-one interactions between the Californios and the Native Americans, (2) many of the Californios were mestizos whose mothers or grandmothers were Native Americans, and (3) lack of pressure on the part of the Californios to suppress Native American beliefs and medicinal practices.

## Background

The migration of people to North America began about 21,000-40,000 years BP over a great land bridge between Siberia and Alaska [1]. Evidence of human settlement dates from about 13,000 years BP on the Channel Islands off the coast of California and from about 10,330 years BP on the mainland near San Luis Obispo ([2]). These early immigrants moved along a coastal route from Alaska either on foot or by boat. Later, Native Americans immigrated to coastal California from inland California and from more eastern areas of North America. They brought with them about 100 languages belonging to seven major language groups [3]. The immigrants also brought with them knowledge of plants used for medicinal purposes gained from the territories they had previously occupied. For example, roots of the species in genus *Rubus* (blackberries) were used to control diarrhea by people in Asia as well as by Native Americans living in different parts of North America [4]. When people immigrated to California, they adopted local species of *Rubus* to combat diarrhea [5]. Once in California, the immigrants adapted new species for medicinal use. The Pomo, for example, used the bark of the California buckeye (*Aesculus californica*), a California endemic, to treat snakebites [6]. Various researchers have examined medicinal use of plants by Native Americans in California since the nineteenth century [7–19]. These studies served as important references in the study reported here.

**Fig. 1:**
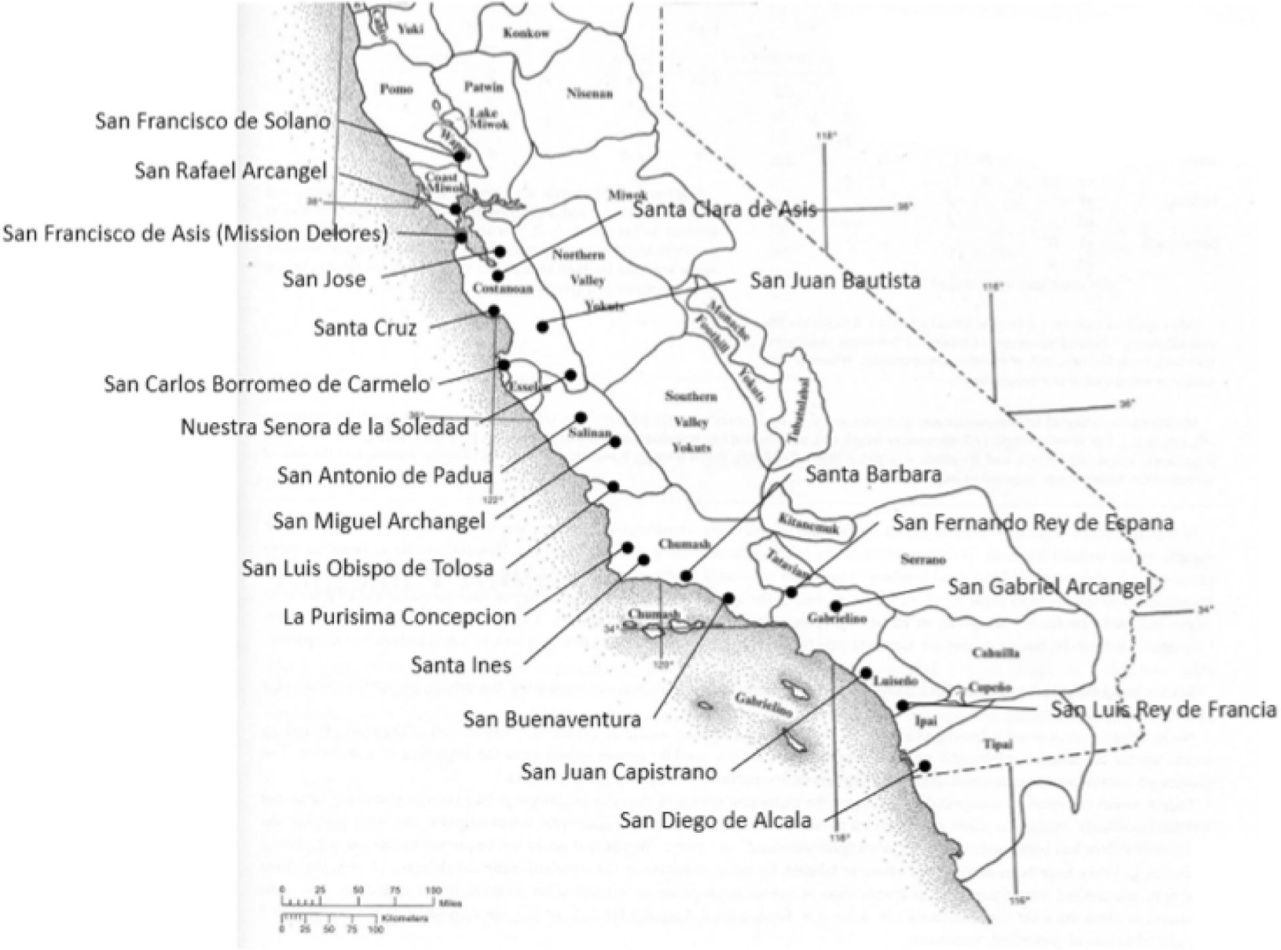
Locations of California Missions and Native American tribal territories

The culture and economy of Native Americans was changed significantly beginning in 1769 with the European colonization of California. An integral part of the Spanish colonization process was the establishment of a system of Missions (Fig. 1). The first Mission was located in what was to become the city of San Diego. Subsequently, Franciscan priests supported by the military moved northward along the California coast to establish a total of 21 Missions [20]. These Missions were established to christianize the Native Americans and to prepare them to serve as a peasant class in the new Spanish territory [21].

In the early Mission period, the priests staffing the Missions were mostly from Spain. The Franciscan priests who established and staffed Missions came primarily from Spain [22, 23]. Thirty-six (72%) of the priests came from northern Spain (Basque territory and the adjacent provinces, Navarra mainly), one from central Spain (2%), and none from the south of Spain. The remaining priests were from Mallorca (8 individuals, 16%) and Mexico (5 individuals, 10%). The Basque territory, Navarra, and Mallorca were the homelands of one-half of the priest at the early California Missions. These priests brought with them knowledge of medicinal herbs used in their homelands. They also brought seeds and cuttings of plants [21] used for medicinal purposes in Mexico and Spain [24].

The California Missions were under the control of Spain from 1769 to 1821. During this time the Native Americans who were converted to christianity at the Missions were known to as *neophytes*. The medical care of the *neophytes* was one of the responsibilities of the priests. The *neophytes*, not being immune to European diseases, succumbed in large numbers to epidemics of measles and smallpox [25]. Contagious native ailments (e.g., colds, dysentery) also spread among the *neophytes* due to their congregation in large numbers at the Missions. The priests responded to the increasing numbers of sick *neophytes* by establishing hospitals at many of the Missions. Although there was a significant power imbalance between the priests and the *neophytes*, the situation called for a sharing of information about medicinal herbs and the employment of neophytes in the treatment of the sick. *Enfermeros* (*neophytes* selected by the priests to serve as nurses) were assigned to care for the sick in these hospitals. The *enfermeros* used medicinal herbs and Spanish medicine to treat the neophytes. Medicinal herbs used by the Native Americans were collected from around the Missions [21], while Spanish medicinal supplies were shipped periodically to California from Mexico [7]. The quantity of medicinal supplies imported from Mexico often became inadequate to treat the increasing number of *neophytes* succumbing to both native and exotic diseases. At times of shortages of medical supplies, the priests and *enfermeros* exchanged knowledge of medicinal plants to broaden the supply of medicines to treat the sick [26]. *Neophytes* were sometimes dispatched by the priests to collect medicinal plants from the wild (Engelhardt 1922).

During the Mission period, seeds of plants for the mission gardens periodically arrived via ships from Europe, South America, and Mexico. Walled gardens, known as *huertas*, were an essential part of the Mission landscapes. They provided growing space for food plants, as well as trees, flowers, and medicinal herbs. Plants grown in the *huertas* were used by both the priests and the Native Americans. The importation of seeds and other goods was curtailed after 1810 when shipping from Spain and the Spanish colonies in the New World was interrupted by the rebellion in Mexico [21]. Mexico gained its independence from Spain in 1821. Following the Mexican rebellion, the independent Mexican government exerted its authority over the Missions. The Mexican authorities attempted to expel the Franciscan priests from the Missions, sell or transfer Mission lands to Mexican citizens, and convert the Mission churches to local parish churches. This process was known as “secularization.” Some missions were abandoned while others assumed the role of parish churches. Mission in more remote locations in California still housed limited number of Native American *neophytes*, but most *neophytes* were transferred to nearby ranches during the Mexican period (1821-1848) were they worked as laborers. Some Native Americans were paid modest salaries for their labor, while most worked for food and a place to live. Individual Native American families and extended families lived on the ranches. A striking contrast to the hundreds who had resided at the missions. The relocation of Native Americans to local ranches provided an opportunity for the sharing of information concerning medicinal plants between the Native Americans and the Californios.

The secularization period ended in 1848 with the annexation of California by the USA following the war with Mexico. Following the annexation, most of the Missions were abandoned and began to fall into disrepair. Without active parishes to maintain the Missions, the old buildings fell prey to the weather. Their roofs gave way first, exposing the soluble adobe walls to the rain. Many of the old buildings were abandoned as unsafe or unsalvageable, many were torn down. For many decades the decay of buildings at the Missions, the missions continued until citizens began to take an interest in them and to propose their restoration. Old records, drawing, and photographs were studied to perform reconstruction of historic buildings, patios, and gardens. At several Missions, medicinal plants were incorporated into the restored gardens.

The purpose of this study is to examine the exchange of medicinal plant information at the California Missions during the Mission and post-Mission periods. Specifically, the exchange between the Native Americans and the priests during the Mission period and the exchange between the Native Americans and the Californios during and following the secularization of the Missions. We hypothesize that an exchange of information on medicinal plants can be identified by comparing the numbers of taxa from Spain that were introduced into California and adopted for use by the Native Americans and the number of taxa from California that were introduced into Spain and adopted by Spanish citizens for medicinal purposes. Furthermore, the exchange of information concerning medicinal plants between the Native Americans and the Californios can be identified by the number of medicinal taxa from Spain and Mexico that were introduced into California and used by the Native Americans and the number of California taxa adopted for medicinal use by the Californios.

## Methods

Two methods were employed in this study: (1) comparison of lists of medicinal plants used by Native American in California before the Mission period, medicinal plants used in Spain, medicinal plants used in Mexico before it gained its independence from Spain, and medicinal plants used by Californios and Native Americans in the post-Mission period and (2) a close reading of diaries, journals, reports, and books written by (i) first-hand observers during the Mission and post-Mission periods and, (ii) modern anthropologists, ethnobotanists, and historians to find accounts of the sharing of information about medicinal plants and to identify reasons why an exchange of information may or may not have taken place.

The lists of medicinal plants and their uses were assembled from a number of sources (Table 1) for the pre- and Mission period (before and during colonization) and the post Mission Period (during and after secularization).

**Table 1:** Bibliographic sources used to assemble the lists of medicinal plants used in different areas

Area	Source
California (Native Americans)	Barrows [27]
	Bean and Saubel [8]
	Faber and Lasagna [28]
	Heinsen [29]
	Lightfoot and Parrish [30]
	Mead [31]
	Timbrook [18]
	Wilken-Robertson [32]
Spain	Akerreta et al. [33, 34]
	Alarcón et al. [35]
	Carrió and Vallès [36]
	Cavero et al. [37, 38]
	Menendez-Baceta et al. [39]
Mexico (Viceroyalty of New Spain)	Argueta and Gallardo [40]
	Heinrich et al. [41]
	Simpson [42]
California (Californios)	Beebe and Senkewicz [43]
	Weber [19]

The data provided were grouped into 14 categories depending on the pathology they treated [37, 38, 44]: (1) cardiovascular diseases; (2) depurative; (3) dermatology; (4) digestive or gastrointestinal problems; (5) metabolic syndromes; (6) infections; (7) skeleto-muscular system; (8) nervous system; (9) sens (eye and ear problems); (10) gynecology; (11) respiratory complaints; (12) urology; (13) ritual procedures; (14) various other ailments (Table 2). Botanical family classification and nomenclature for species names were authenticated according to Hickman [45], Stevens [46] and [47] (www.ipni.org).

**Table 2:** Classifying diseases

Number	Categories	Affection
1	CAR: Cardiovascular diseases	Antivaricose, blood disorders, blood pressure regulator (thick blood, antihypertensive), cardiotonic (heart problems), clean the blood, external hemostatic, hemorrhoids (piles), high cholesterol, phlebitis, uric acid, vasotonic (circulatory problems, enhance circulation)
2	DEP: Diuretic, laxative, diaphoretic	Clean the body, depurative, fluid retention
3	DER: Dermatology	Acne, anti-ecchymotic, baldness (hair loss), bites (dog, snake, insect, nettle stings), blisters and grazes, boils; bruises, burns, calcanean spurs, calluses or corns, cellulitis, chilblains, clean the skin, eczema, embedded thorns, gangrene, hard skin, mouth infections and ulcers, pruritus, psoriasis, skin disorders (infection, inflammation, rash), ulcers; vulnerary, warts, whitlows, wounds and cuts (infection)
4	GAS: Digestive or gastrointestinal problems	Antiemetic, antihelminthic, appetizer (tonic), carminative (gases), clean the stomach, constipation (laxative), diarrhea, digestive disorders, emetic, gall stones, gastritis (gastric anti-inflammatory), heartburn, internal ulcers, intestinal worms, liver disorders (clean, inflammation, jaundice, protection, pain), purgative, stomach pain and disorders, teeth (disorders, strengthening, pain)
5	MET: Metabolic syndromes	Allergic reactions, anti-inflammatory, diabetes, hypoglycemic, metabolic disorders, salutiferous, stimulate immune system
6	INF: Infections	Antiherpes, fever (antipyretic), internal antiseptic (infections)
7	SKE: Skeleto-muscular system	Antialgic muscular, antispasmodic, arthrosis, body pains, broken bones, decalcifications, lumbago, muscle anti-inflammatory, muscular and joint pains, musculoskeletal disorders, osteoarthritis (arthritis), rheumatism (antirheumatic), sciatica, sprains.
8	NER: Nervous system	Analgesic, antiparkinsonian, depression, headache, insomnia, nervousness, relaxant, sadness, sedative (tranquilizer), sickness, stimulant
9	SEN: Sens	Eyes (clean, conjunctivitis, antiseptic, inflammation, irritation, pain, rheum, sties, visual protector), ear (disorders and pain)
10	GYN: Gynecology	Abortive, dysmenorrhea, anti-metrorrhagic, emmenagogue, galactofugue, galactogenous, menstruation, premenstrual pain, puerperium antiseptic, tonic after give birth, vaginal infections.
11	RES: Respiratory complaints	Anticatarrhal, antitussive, asthma, bronchitis, chest infections, cold, cough, expectorant (mucolytic) flu, hoarseness, inflammation, influenza, pharyngeal problems, phlegm; pneumonia, sinusitis, sore throat, tuberculosis, whooping cough
12	URO: Urology	Cystitis, kidney disorders (stones and clean), masculine impotence, prostate inflammations and disorders, renal anti-inflammatory, litothriptic and protector, urinary antiseptic and retention
13	RIT: Ritual procedures	To protect from illness and bad spirits
14	VAR: Various	Undefined pain and illnesses (anemia, antiscorbutic, diaphoretic, general malaise and pains, healthy, iron- deficiency, panacea, to give up alcohol, and vitamin)

To determine if any California species were introduced in Spanish and/or European botanical gardens a literature review was carried conducted [48–54]. Several databases were also consulted: www.floraiberica.es; www.fitoterapia.net [55–57];.

A comparison of the assembled lists identified medicinal plant taxa that were used in two different areas (e.g., California and Spain). If taxa native to California were reported to be used in present-day Spanish medicinal gardens, then we assumed information of the medicinal use of these plants had been shared between the Native Americans and the Spanish priest. Likewise, if taxa native to Spain were present in herb gardens at the Missions or reported to have been used by Native Americans during the post-Mission period, we assumed that sharing of knowledge had taken place.

## Results

**Fig. 2:**
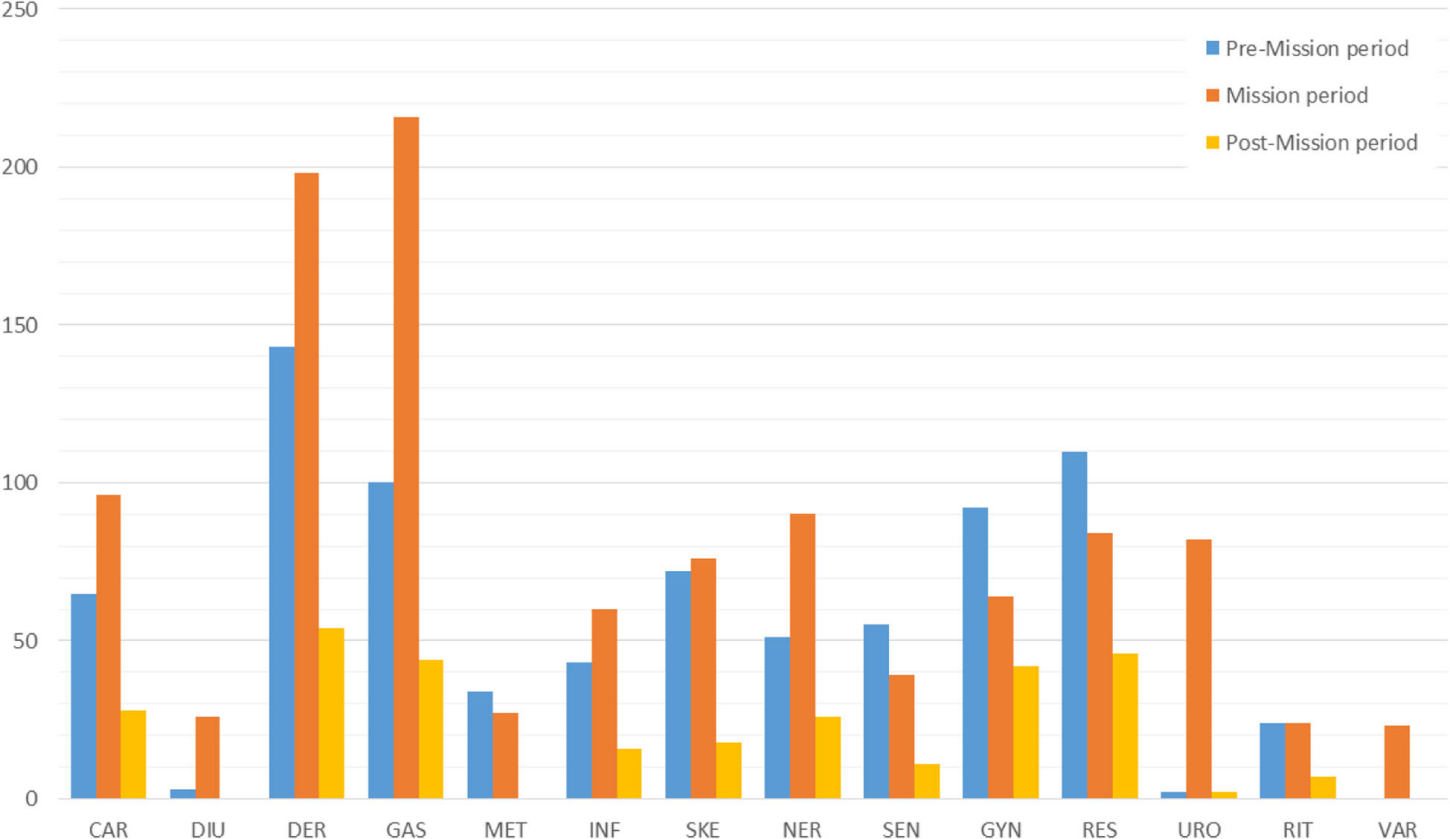
Therapeutical categories of medicinal plants

A total of 822 taxa belonging to 136 botanical families were identified (Table 3). Seven hundred twelve of them had been used during pre- and Mission Period; 265 of them were plants used by Native Americans in California before colonization, 448 taxa were used for medicinal purposes in Spain or in Mexico (Table 3). The most commonly used plants were employed to treat sores, wounds, and skin problems, for respiratory diseases, gastrointestinal tract problems, reproductive affections, and cardiovascular diseases (Fig. 2). The preparation and application of plant materials for medicinal purposes by the Native Americans in California included the direct application of leaves to the affected area (e.g., *Rhamnus californica* Eschsch.—treat rheumatism); drinking water in which the plant material had been boiled (e.g., *Rubus ursinus* Cham. & Schldl—treat diarrhea); application of a poultice prepared from the plant material (e.g., *Malva parviflora* L.—treat wounds), eating the plant or plant part (e.g., *Rorippa nasturtium* (L.) Hayek—treat liver ailments), bathing the skin with water in which to plant had been boiled (e.g., *Wyethia helenioides* (DC.) Nutt.—treat sores); rubbing dry ashes of a plant on the skin (e.g., *Scripus californicus* (C. Mewyer) Steudel—treat poison oak); chewing plant parts (e.g., *Lomatium californicum* (Torrey and Gray) J. Coulter & Rose—treat pain).

**Table 3:** Medicinal plants used before, during and after the Mission period, and present time at Mission Gardens. The numbers refer to emic and etic illness groupings (see Table 2)

Botanical family*	Medicinal plants*	Native	Pre-Mission period	Mission period	Post-Mission period
*Acanthaceae*	*Acanthus mollis* L.	Europe		3	
	*Justicia spicigera* Schltdl.	Mexico		3, 4	
*Adoxaceae*	*Sambucus ebulus* L.	Eurasia		3	
	*Sambucus* sp.	California	3, 11		3, 6, 10
	*Sambucus mexicana* C. Presl [*S. nigra* L. ssp. *caerulea* (Raf.) R. Bolli]	California, Mexico	1, 3, 4, 6, 7, 8, 10, 11	7, 10	8, 11
	*Sambucus nigra* L. ssp. *nigra*	Europe, Africa		1, 2, 3, 4, 6, 7, 8, 9, 10, 11, 12	
*Agavoideae*	*Agave* sp*.*	California and Mexico		4	
	*Agave americana* L.	Mexico, USA introduced from Europe		11	
*Amaranthaceae*	*Amaranthus hybridus* L.	Eastern U.S.A. introduced from Europe		1, 3, 4	
	*Atriplex* sp.	California	10		
	*Atriplex lentiformis* (Torrey) S. Watson	California	3, 9, 10		
	*Beta vulgaris* L. var. *conditiva* Alef.	Eurasia, Africa		1	
	*Beta vulgaris* L. var*. maritima* (L.) Moq.	Eurasia, Africa		5	
	*Chenopodium ambrosioides* L. [*Dysohania ambrosioides* (L.) Mosyakin & Clemants]	Mexico		4	3
	*Chenopodium californicum* (S. Watson) S. Watson	California	4, 8, 10		
	*Chenopodium graveolens* Willd.	Mexico		4, 10	
	*Chenopodium rubrum* L. [*Oxybasis rubra* (L.) S. Fuentes, Uotila & Borsch]	California			
	*Dysphania ambrosioides* (L.) Mosyakin & Clemants	Mexico			3
	*Dysphania botrys* (L.) Mosyakin & Clemants	Europe			3
	*Iresine celosia* L.	Mexico		3, 6, 12	
*Amaryllidaceae*	*Allium* sp.	California	3, 4, 11		10, 11
	*Allium cepa* L.	Asia introduced from Europe		1, 3, 4, 11, 12	
	*Allium porrum* L.	Europe		1, 4, 11	
	*Allium sativum* L.	Asia introduced from Europe		1, 3, 4, 5, 6, 7, 8, 11	6, 11, 12
*Anacardiaceae*	*Mangifera indica* L.	India		4, 9	
	*Pistacia lentiscus* L.	Mediterranean region		3, 4	
	*Rhus aromatic* L. (*R. trilobata* Nutt.)	California	4		
	*Rhus ovate* S. Watson	California	1, 10		
	*Schinus molle* L.	South America		4, 6, 7, 9, 10	
	*Spondias purpurea* L.	Mexico		4, 6, 9, 10	
	*Toxicodendron diversilobum* (Torrey & A. Gray) E. Greene	California	1, 3, 4, 6, 9		3
	*Toxicodendron venenosum* (S. Watson) Rydb. var. *venenosum* (*Zigadenus venenosus* S. Watson)	California	3		
*Anacariaceae* *Annonaceae*	*Malosma laurina* (Nutt.) Abrams	California	6		
	*Annona cherimola* Mill.	South America		4, 6	
	*Annona reticulate* Linn.	Mexico		3, 4	
*Apiaceae*	*Angelica* sp.	California	3, 4, 7, 8		
	*Apium graveolens* L.	Europe		1, 2, 11, 12	4
	*Aralia californica* S. Watson	California	3		
	*Carum carvi* L.	Europe, naturalized in California			
	*Coriandrum sativum* L.	Europe, naturalized in California			8
	*Crithmum maritimum* L.	Eurasia, Africa		2, 4, 14	
	*Daucus carota* L.	Eurasia		1, 3, 9	
	*Daucus pusillus* Michaux	California	1, 3, 8, 10, 11		1, 8, 10, 11
	*Eryngium campestre* L.	Eurasia		2, 10	
	*Foeniculum vulgare* Mill.	Europe		1, 2, 3, 4, 10, 11	4
	*Hedera helix* L.	Europe		3, 4, 10	3, 10
	*Lomatium californicum* (Torrey & A. Gray) Mathias & Constance (*Leptotaenia californicum* Nutt.)	California	4, 7, 8, 10, 11		
	*Lomatium utriculatum* (Torrey and Gray) J. Coulter & Rose	California			11, 13
	*Petroselinum crispum* (Mill.) Fuss	Europe		2, 3, 4, 5, 8, 9, 10, 14	4, 13
	*Pimpinella anisum* L.	Asia Minor, introduced from Europe		4	
	*Sanicula arguta* J. Coult. & Rose	California	5		
*Apocynaceae*	*Gonolobus niger* (Cav.) R. Br.	Mexico		6	
	*Nerium oleander* L.	Europe			
	*Plumeria rubra* L.	Mexico		3, 4, 6	
	*Stemmadenia donnell-smithi* Woodson	Europe		3	
	*Thevetia thevetioides* (Kunth) Schum.	Mexico		3	
	*Vinca difformis* Pourr.	Europe		10	
*Aquifoliaceae*	*Ilex aquifolium* L.	Europe		3	
*Araceae*	*Arisarum vulgare* Targ.-Tozz.	Eurasia, Africa		3	
	*Arum italicum* Mill.	Mediterranean region		3, 7	
*Arecaceae*	*Chamaerops humilis* L.	Europe		3, 4	
	*Cocos nucifera* L.	Malaysia			6
*Aristolochiaceae*	*Aristolochia maurorum* L.	Mexico		8	
	*Aristolochia monticola* Brandegee	Mexico			3, 4
	*Aristolochia pentandra* Jacq.	Mexico		3	
	*Asarum caudatum* Lindl.	California	3, 7		
*Asclepiadaceae*	*Asclepias* sp.	California	4		
	*Asclepias curassavica* L.	Mexico		3, 9	9
	*Asclepias eriocarpa* Benth.	California	3, 4, 10		
	*Asclepias lemmonii* A. Gray	Mexico, South West USA			11
*Asparagaceae*	*Asparagus acutifolius* L.	Mediterranean region		2	
	*Asparagus horridus* L. in J.A.Murray	Europe		12	
	*Camassia* sp.	California	3, 7		
	*Chlorogalum pomeridianum* (DC.) Kunth	California	2, 3, 5		9, 11
	*Maianthemum racemosum* (L.) Link [*Smilacina racemosa* (L.) Link]	California	7		
	*Ruscus aculeatus* L.	Eurasia, Africa		1	
	*Urginea maritima* (L.) Baker	Eurasia, Africa		7	
	*Yucca baccata* Torrey	California	3		
	*Yucca schidigera* Roezl ex Ortgies	California, Mexico		1, 3, 4, 7, 8	
*Asphodelaceae*	*Aloe* sp.	California		1, 3, 4	
	*Aloe maculata* All.	Africa introduced from Europe		3, 7	
	*Aloe vera* (L.) Burm. fil.	Asia introduced from Europe		3	
*Aspleniaceae*	*Asplenium trichomanes* L. ssp. *trichomanes*	Eurasia, California		10, 11	
	*Ceterach officinarum* Willd.	Eurasia, Mediterranean region		1, 4	
*Asteraceae*	*Acamptopappus sphaerocephalus* (A. Gray) A. Gray	California	11		
	*Achillea* sp.	California	9, 11		
	*Achillea ageratum* L.	Europe		6, 8	
	*Achillea millefolium* L. ssp. *millefolium*	California, Europe	1, 3, 9, 10, 11	1, 3, 4, 7, 8, 10, 11	
	*Acourtia microcephala* DC. [*Perezia microcephala* (DC) A. Gray]	California	1, 3, 4, 10, 11		
	*Ageratina* sp.	California			3, 11
	*Ageratina adenophora* (Spreng.) R.M.King & H.Rob.	Mexico			3, 11
	*Ambrosia monogyra* Torr. & Gray	California	3, 11		
	*Ambrosia pilostachya* DC.	California	3, 7		
	*Amphipterygium adstringens* (Schltdl.) Schiede ex Standl.	Mexico		1, 4, 7	
	*Anacyclus clavatus* Pers.	Europe		4, 8	
	*Anthemis arvensis* L. *ssp. arvensis*	Eurasia, Africa		1, 4, 8, 9	
	*Arctium minus* Bernh.	Europe		3	7
	*Artemisia* sp.	California	4, 5, 7, 9, 10, 11		
	*Artemisia abrotanum* L.	Eurasia, Africa		3	
	*Artemisia absinthium* L.	Europe			4
	*Artemisia alba* Turra	Europe		4	
	*Artemisia californica* Less.	California	3, 5, 7, 8, 9, 10, 11		
	*Artemisia cana* Pursh ssp. *bolanderi* (A. Gray) G. Ward	California			1, 3, 8
	*Artemisia douglasiana* Besser (*A. heterophylla* Nutt.)	California	1, 3, 4, 5, 6, 7, 8, 9, 10, 11		
	*Artemisia drancunuloides* L.	California, Europe	1, 4, 6, 7, 8, 9, 11		8
	*Artemisia herba-alba* Asso	Europe		11	
	*Artemisia mexicana* Willd.	Mexico and South West USA		4, 7	
	*Artemisia ludoviciana* Nutt.	California, Mexico	3, 8, 10, 11	4, 6, 10, 11	4
	*Artemisia pycnocephala* DC	California			
	*Artemisia tridentate* Nutt	California	3, 4, 8, 10, 11		
	*Baccharis glutinosa* Pers. [*B. salicifolia* (Ruiz Lopez & Pavon) Pers.]	California, Mexico	3, 9, 11, 13	3, 4, 11	
	*Baccharis pilularis* DC.	California	3, 11		
	*Baccharis plummerae* A. Gray	California	8, 13		
	*Baccharis pteronioides* A. Gray	Mexico, South West USA			3, 7, 8, 11
	*Baccharis sarothroides* A. Gray	California		7, 10	
	*Balsamorhiza sagittalta* (Pursh) Nutt.	California	4, 6, 8, 10, 11		8, 10, 11
	*Bidens aurea* (Aiton) Sherff	Mexico		4	
	*Calea urticifolia* (Mill.) DC.	Mexico		6, 12	
	*Calea zacatechichi* Schltdl. (*C. ternifolia* Kunth.)	Mexico		4, 12	
	*Calendula arvensis* L.	Europe		3, 8, 10, 11	
	*Calendula officinalis* L.	Europe, naturalized in California		3	
	*Carduus pynocephalus* L. ssp. *pynocephalus*	Europe		3	
	*Carlina acanthifolia* All. ssp. *cynara* (Pourret ex Duby) Rouy	Europe		13	
	*Centaurea aspera* L.	Europe		1, 5	
	*Chamaemelum nobile* (L.) All.	Europe, naturalized in California		2, 3, 4, 8, 9, 10, 11, 14	
	*Chamomilla recutita* (L.) Rauschert (*Matricaria chamomilla* L.)	Europe, naturalized in California		4	
	*Chamomilla suaveolens* (Pursh) Rydb. (*Matricaria discoidea* DC.)	Europe, naturalized in California	3, 4, 5, 6, 7, 10, 11		
	*Chaptalia nutans* (L.) Polak.	Caribbean		3, 7	
	*Chrysanthemum balsamita* L. (*Tanacetum balsamita* L.)	Europe, naturalized in California			
	*Cichorium intybus* L.	Europe, naturalized in California			1, 4
	*Cirsium* sp.	California	3, 4, 10		
	*Cirsium arvense* (L.) Scop.	Europe, naturalized in California		3	
	*Conyza canadensis* (L.) Cronq. (*Erigeron canadensis* L.)	California	8, 12		
	*Corethrogyne filaginifolia* (Hook. & Arn.) Nutt.	California	1, 10, 11		
	*Cynara scolymus* L.	Mediterranean region		4, 5	
	*Deinandra fasciculate* (DC.) Greene [*Hemizonia fasciculata* (DC.) Torr. & A. Gray]	California	8		
	*Encelia californica* Nutt.	California			
	*Encelia farinose* Torrey & A. Gray	California	1, 9		
	*Ericameria arborescens* (A. Gray) E. Greene	California	1, 3, 4, 5, 7, 9, 10, 11		
	*Ericameria laricifolia* (A. Gray) Shinn.	California	11		
	*Ericameria nauseosa* (Pall. Ex Pursh) G. L. Nesom & Baird [*Bigilovia nauseosa* M. E. Jones; *Chrysothamnus nauseosa* (Pall. Ex Pursh) Britton]	California	1, 9, 10		
	*Ericameria palmeri* (A. Gray) H. M. Hall var. *pachylepsis* (H. M. Hall) G. Nesom [*E*. *acradenius* (Greene) S. F. Blake; *Haplopappus palmeri* A. Gray; *Aplopappus palmeri* Gray]	California	3, 10, 11		
	*Erigeron canadensis* L. [*Conyza canadensis* (L.) Cronq.)	California	4, 8, 13		
	*Erigeron foliosus* Nutt. var. *foliosus* (*E. foliosus* Nutt. var. *stenophyllus; E. utahensis* Gray)	California	11		
	*Erigeron karwinskianus* DC.	Mexico		4, 6, 14	
	*Eriophyllum co*nfertiflorum (DC.) A. Gray	California	7		
	*Eupatorium perfoliatum* L.	Eastern USA			3, 11
	*Franseria ambrosioides* (Cav.) Payne	California		3, 7	
	*Gnaphalium* sp.	California		10, 12	
	*Gnaphalium bicolor* Bioletti [*Pseudognaphalium bioletti* (Bioletti) A. Anderb.]	California	1, 4		
	*Gnaphalium canescens* DC. [*Pseudognaphalium canescens* (DC.) W.A. Weber]	California	1, 3, 4, 10, 11		
	*Grindelia camporum* E. Greene (*G. robusta* Nutt.)	California	1, 3, 10, 11		
	*Grindelia hirsutala* Hook. & Arn.	California	10		
	*Grindelia stricta* DC. (*G. latifolia* Kellogg)	California	3		
	*Gutierrezia microcephala* (DC.) A. Gray	California	9		
	*Helenium mexicanum* Kunth	Mexico		10	
	*Helenium puberulum* DC.	California	3, 6, 10, 11		
	*Helianthus annuus* L.	Europe		3, 8	
	*Helichrysum italicum* G. Don f.	Mediterranean region		4	
	*Helichrysum stoechas* (L.) Moench spp*. stoechas*	Mediterranean region		1, 4, 8, 11	
	*Heterotheca grandiflora* Nutt.	California	3, 6		
	*Heterotheca inuloides* Cass.	Mexico		3, 10	
	*Inula montana* L.	Western mediterranean		3	
	*Inula viscosa* (L.) Ait.	Mediterranean region		3	
	*Jasonia glutinosa* (L.) DC*.*	Europe, Africa		4, 8, 14	
	*Jasonia tuberosa* (L.) DC.	Europe		3, 4, 7, 11	
	*Leptosyne maritime* (Nutt.) A. Gray	California	4		
	*Madia sativa* Molina	California	7, 10		7
	*Matricaria discoidea* DC.	Asia, North West USA		4, 8	4, 6, 7, 10
	*Matricaria recutita* L.	Europe			
	*Mikania* sp.	Mexico and West USA		4, 12	
	*Montanoa tomentosa* Cerv.	Mexico		7	
	*Onopordum acanthium* L.	Eurasia		3, 4	
	*Parthenium hysterophorus* L.	Mexico			
	*Phagnalon saxatile* (L.) Cass.	Mediterranean region		2	
	*Pleiacanthus spinosus* (Nutt.) Rydb. (*Lygodesmia spinosa* Nutt.)	California	2		
	*Polymnia maculata* Cav.	Mexico		3, 4, 6	
	*Pseudognaphalium californicum* (DC.) Anderb. (*Gnaphalium decurrens* E. Ives)	California	4, 8, 10, 11		
	*Pseudognaphalium canescens* (DC.) W. A. Weber [*Gnaphalium canescens* DC.]	California	7		
	*Santolina chamaecyparissus* L.	Europe		3, 4, 8	
	*Santolina chamaecyparissus* L. ssp. *squarrosa* (DC.) Nyman	Europe		1, 4, 8, 9, 11	
	*Santolina chamaecyparissus* L. ssp. *magonica* O.Bolòs, R.Mol. et P.Monts. var. *teucrietorum* O.Bolòs et Vigo	Europe		3, 4, 7, 8, 9, 10, 11, 12	
	*Senecio angulifolius* DC.	Mexico		3, 9	
	*Senecio flaccidus* Less. var. *douglasii* (DC.) B.L. Turner & T.M. Barkley (*S. douglasii* DC.)	California	3, 6, 7, 10, 13		
	*Solidago californica* Nutt. [*S. velutina* DC. ssp. *californica* (Nutt.) Semple]	California	3, 4, 5, 9, 10, 11		
	*Sonchus asper* (L.) Hill	Eurasia, Africa		3	
	*Sonchus oleraceus* L.	Eurasia		3	
	*Sonchus tenerrimus* L.	Europe, Africa, Middle East		4	
	*Tagetes erecta* L.	Mexico		4, 6, 9, 10, 12	
	*Tagetes lucida* (Sweet) Voss	Mexico		3, 4, 7, 12	
	*Tanacetum balsamita* L.	Europe		3, 4	
	*Tanacetum corymbosum* (L.) Sch. Bip.	Europe		4	
	*Tanacetum parthenium* (L.) Sch. Bip.	Eurasia		4, 8, 13	
	*Tanacetum vulgare* L.	Europe		4, 8	4
	*Taraxacum officinale* Weber	Europe		1, 3, 4, 12	
	*Thelesperma gracile* (Torr.) A. Gray [*T. megapotamicum* (Spreng.) Kuntze]	Mexico and South West USA		4, 8	
	*Tithonia diversifolia* (Hemsl.) A. Gray	Mexico		3, 6, 9	
	*Trixis californica* Kellogg	California and Mexico		3, 9	
	*Tussilago farfara* L.	Eurasia		3, 11	
	*Verbesina* sp.	California			3
	*Wyethia angustifolia* (DC.) Nutt.	California	3, 10		
	*Wyethia helenioides* (DC.) Nutt.	California	3, 5, 9, 10		
	*Xanthium strumarium* L.	California	3,13		
*Begoniaceae*	*Begonia heracleifolia* Cham. & Schltdl.	Mexico		3, 4	
*Berberidaceae*	*Berberis aquifolium* Pursh	California	11		
	*Berberis nevinii* A. Gray	California			
*Betulaceae*	*Alnus* sp.	California	3, 4		
	*Alnus arguta* (Schltdl.) Spach	Mexico		3, 12	
	*Betula occidentalis* Hook.	California			4, 10, 11
	*Betula pendula* Roth	Europe		2	
	*Corylus cornuta* var. *californica* (A. DC.) E. Murray	California			11
*Bignoniaceae*	*Crescentia cujete* L.	Mexico		3, 4	
	*Parmentiera edulis* DC.	Mexico		9, 10, 12	
	*Tabebuia rosea* (Bertol.) DC.	Mexico		3, 7, 12	
*Bixaceae*	*Bixa orellana* L.	Mexico		3, 6	
	*Cochlospermum vitifolium* (Willd.) Spreng.	Mexico		3, 4	
*Boraginaceae*	*Borago officinalis* L.	Europe, naturalized in California		1, 2, 3.6, 11	1, 4, 10
	*Cordia curassavica* (Jacq.) Roem. & Schult.	Mexico		3, 5, 8, 10	
	*Ehretia tinifolia* L.	Mexico		12	
	*Eriodictyon californicum* (Hook. & Arn.) Torrey	California	1, 3, 4, 6, 7, 8, 10, 11		8, 9, 10
	*Eriodictyon crassifolium* Benth.	California	1, 3, 7, 10, 11		
	*Eriodictyon trichocalyx* A. Heller	California	1, 3, 6, 7, 10, 11		
	*Heliotropium curvassavicum* L. var. *oculatum*	California	5		
	*Lithospermum officinale* L.	Europe		4	
	*Phacelia distans* Benth.	California			1, 3, 4, 10
	*Phacelia ramoisissima* Lehm.	California	6, 10, 11		
	*Pulmonaria longifolia* (Bast.) Boreau	Europe		11	
	*Symphytum asperum* Lepech.	Asia, introduced from Europe			1, 8, 10
	*Symphytum officinale* L.	Europe		7	
	*Symphytum tuberosum* L. ssp. *tuberosum*	Europe		7	
	*Tournefortia hartwegiana* DC.	Mexico		4, 7, 10, 13	
*Brassicaceae*	*Brassica nigra* (L.) Koch	Eurasia, Africa		11	
	*Brassica oleracea* L. ssp. *oleracea*	Europe		3, 4, 8	
	*Brassica rapa* L.	Europe, Asia			7, 10
	*Capsella bursa-pastoris* (L.) Medik.	Eurasia		1, 4, 5, 10	
	*Coronopus didymus* (L.) Sm.	South America		1, 6, 11	
	*Coronopus squamatus* (Forsk.) Asch.	Mediterranean region		1, 2	
	*Descurainia pinnata* (Walter) Britton [*Sisymbrium canescens* (Phil.) Reiche., *S. pinnatum* (Walter) Britton]	California	4		
	*Lepidium latifolium* L.	Eurasia		12	
	*Lepidium nitidum* Torrey & Gray	California	4, 6		
	*Raphanus raphanistrum* L. ssp. *sativus* (L.) Domin	Europe			3
	*Rorippa* sp.	California	11		
	*Rorippa nasturtium*-*aquaticum* (L.) Hayek (*Nasturtium officinalis* W. T. Aiton)	Europe, Asia, naturalized in California and Mexico	1, 4	1, 6, 12, 14	
*Burseraceae*	*Bursera grandifolia* (Schltdl.) Engl.	Mexico		4, 8, 12	
	*Bursera microphylla* A. Gray	California	3		
	*Bursera simaruba* (L.) Sarg.	Mexico		3, 12	
	*Protium copal* Engl.	Mexico		1, 3, 4, 7	
*Buxaceae*	*Buxus balearica* Lam.	Eurasia, Africa		4	
	*Buxus sempervirens* L.	Europe		4	
*Cactaceae*	*Cylindropuntia acanthrocarpa* (Engelm. & Bigelow) F. M. Knuth (*Opuntia acanthrocarpa* Engelm. & Bigelow)	California	3		
	*Lemaireocereus thurberi* (Engelm.) Britton & Rose [*Stenocereus thurberi* (Engelm.) Britton & Rose]	Mexico		1, 3, 12	
	*Lophophora williamsii* (Lem.) J. M. Coult.	Mexico, Texas		3	1, 4
	*Opuntia maxima* A.Berger	Mexico introduced from Europe		1, 2, 3, 4, 5, 11, 12	
	*Opuntia* sp.	California and Mexico	1, 3, 7	1, 4	10
	*Opuntia imbricate* DC*. [Cylindropuntia imbricate* (DC.) Haw.*]*	Mexico		4, 10	
	*Opuntia leucotricha* DC.	Mexico		1, 4, 12	
	*Opuntia tuberosus* (Pfeiff.) Britton & Rose	California and Mexico		8	
*Cannabaceae*	*Cannabis sativa* L.	Eastern Asia			
	*Humulus lupulus* L.	Europe		8	
*Caprifoliaceae*	*Lonicera* sp.	California	7, 10		
	*Lonicera implexa* Ait.	Europe		1, 3, 5	
	*Lonicera interrupta* Benth.	California	3, 9, 10		
	*Lonicera subspicata* Hook. & Arn.var. *subspicata*	California	3, 10		
	*Scabiosa* sp.	Europe			1
	*Valeriana officinalis* L.	Europe			
*Caricaceae*	*Carica papaya* L.	Europe		3, 4, 10	
*Caryophyllaceae*	*Herniaria hirsuta* L. ssp. *cinerea* (DC. in Lam. et DC.) Arcang.	Eurasia, Africa		2, 8, 12	
	*Paronychia argentea* Lam.	Mediterranean region		1	
	*Silene laciniata* Cav. ssp. *major* C. Hitchc. & Maguire (*S*. *laciniata* Cav. ssp. *laciniata*)	California	5, 7		
	*Spergularia salina* J. Presl & C. Presl [*S. marina* (L.) Besser]	California			1, 3, 4, 9, 10, 11
	*Stellaria media* (L.) Vill.	Europe			
*Celastraceae*	*Hippocratea excelsa* Kunth	Mexico		3	
	*Torreya californica* Torrey [*Tumion californicum* (Torrey) Greene]	California	4, 8, 11		
*Cistaceae*	*Cistus albidus* L.	Europe. Africa		3, 8, 11	
	*Cistus salviifolius* L.	Eurasia, Africa		3, 12	
*Commelinaceae*	*Commrlina erecta* L.	Mexico		4, 7,12	
	*Rhoeo discolor* (L'Hér.) Hance (*Tradescantia spathacea* Sw.)	Mexico		3, 6, 7	
*Convolvulaceae*	*Cuscuta* sp.	California and Mexico		4, 12	
	*Cuscuta californica* Hook. & Arn.	California	11		
	*Ipomoea arborescens* (Humb. & Bonpl. Ex. Willd.) G. Don	Mexico		1, 4, 8, 12	
	*Ipomoea stans* Cav.	Mexico		4, 8	
*Cornaceae*	*Cornus sericea* L. ssp. *californica* (*C. californica* C.AQ. Meyer)	California	11		
*Crassulaceae*	*Dudleya pulverulenta* (Nutt.) Britton & Rose	California	3, 10		
	*Hylotelephium maximum* (L.) Holub	Eurasia		3, 8	
	*Hylotelephium telephium* (L.) H. Ohba	Eurasia		3	
	*Kalanchoe pinnata* (Lam.) Pers*.*	Madagascar		3	
	*Sedum oxypetalum* Kunth	Mexico		3	
	*Sedum spathulifolium* Hook.	California	3, 10, 11		
	*Sedum spurium* M. Bieb.	Asia introduced from Europe		3	
	*Sempervivum tectorum* L.	Europe		9	
	*Umbilicus rupestris* (Salisb.) Dandy	Europe		3	
*Cucurbitaceae*	*Citrullus lanatus* var. *lanatus* (Thunb.) Matsum. & Nakai	Africa			6, 11
	*Cucumis sativus* L.	Asia, introduced from Europe		4	
	*Cucurbita foetidissima* Kunth	California and Mexico	3, 4, 7, 11	12	3, 7
	*Cucurbita maxima* Duchesne.	South America		4	
	*Cucurbita palmate* S. Wats.	California			4, 11
	*Cucurbita pepo* L.	South America		12	
	*Ibervillea sonorae* S. Wats.	Mexico		6	
	*Luffa aegyptiaca* Mill.	Egypt, introduced from Europe		3	
	*Marah fabacea* (Naudin) Greene	California	3, 13		3
	*Marah macrocarpus* E. Greene	California	1, 3, 4, 5, 7, 9, 11		
	*Momordica charantia L.*	South Indian		4, 8, 12	
*Cupressaceae*	*Hesperocyparis macrocarpa* (Hartw.) Bartel (*Cupressus macrocarpa* Hartw.)	California	7		
	*Juniperus californica* Carr.	California	7, 11, 13		
	*Juniperus chinensis* L.	Asia			
	*Juniperus communis* L.	Eurasia		3, 4, 7, 9	
	*Juniperus deppeana* Steud*.*	Mexico and South West USA		7, 12	
	*Juniperus phoenicea* L.	Mediterranean region		3	
	*Sequoia sempervirens* (D. Don) Endl.	California	3, 9, 10, 11		
	*Taxodium mucronatum* Ten.	Mexico and South West USA		3, 6, 9	
*Cyperaceae*	*Schoenoplectus* sp.	California	3, 7		
	*Scirpus* sp.	California	7		
	*Scirpus acutus* L. var. *occidentalis* (S. Watson) Beetle [*Schoenoplectus acutus* (Muhl. Ex Bigelow) A. Love & D. Love var. *occidentalis* (S. Watson) S. G. Sm.]	California	3, 7		
	*Scirpus californicus* (C. Mewyer) Steudel (*Schoenoplectus californicus* C. A. Mey. Palla)	California	3		
*Datiscaceae*	*Datisca glomerata* (C. Presl) Baillon	California	3, 7, 8, 10		
*Dennstaedtiaceae*	*Pteridium aquilinum* (L.) Kuhn.	California, Europe, Mexico		1	11, 13
*Dioscoreaceae*	*Dioscorea* sp.	Mexico		5, 7, 8	
	*Tamus communis* L.	Europe		3, 7	
*Dryopteridaceae*	*Dryopteris arguta* (Kaulf.) Watt [*Aspidium rigidum* Sw. *arguta* (DC.) Eat.]	California	3, 11		
	*Dryopteris filix-mas* (L.) Schott	Europe, California		4	
*Ebenaceae*	*Diospyros kaki* L.f.	Asia introduced from Europe, California		1, 4	
*Ephedraceae*	*Ephedra* sp.	California	6		
	*Ephedra californica* S. Wats.	California	1, 3, 4, 6, 10, 11, 13		
	*Ephedra viridis* S. Watson	California	1, 3, 4, 6, 11, 13		
*Equisetaceae*	*Equisetum* sp.	California	4, 10, 11, 13		
	*Equisetum arvense* L.	California, Europe, Mexico	3	1, 2, 3, 7, 11, 12	1, 11
	*Equisetum hyemale* L.	Europe, Mexico			1, 11, 12
	*Equisetum laevigatum* A. Braun (*E. funstoni* A. A. Eaton)	California	3, 5, 7, 11, 13		
	*Equisetum ramossissimum* Desf.	Eurasia, Africa		1, 2	
	*Equisetum telmateia* Ehrh.	Eurasia, Africa		1, 3, 7, 12	
*Ericaceae*	*Arbutus unedo* L.	Europe		1	
	*Arbutus menziesii* Pursh	California	3, 4, 10		
	*Arbutus xalapensis* Kunth	Mexico, South West USA		7	
	*Arctostaphylos glauca* Lindl.	California	3, 4, 13	2, 12	
	*Arctostaphylos uva-ursi* (L.) Spreng.	California and Europe		12	3
	*Erica cinerea* L.	Europe		12	
	*Vaccinium* sp.	California	12		
*Euphorbiaceae*	*Acalypha alopecuroidea* Jacq.	Mexico		4	
	*Chamaesyce* sp. (*Euphorbia* sp.)	California	3, 9, 11		
	*Cnidoscolus chayamansa* (Mill.) I. M. Johnst.	Mexico		3, 13, 12	
	*Cnidoscolus urens* L. ssp. *stimulosus* (Michx.) Govaerts	Mexico			11
	*Croton* sp.	Asia		4	
	*Croton californicus* Muell	California	7, 9, 10		
	*Croton draco* Schldtl.	Mexico		9	
	*Croton fragilis* Schltr.	Mexico		4, 6	
	*Croton setiger* Hook. [*Eremocarpus setiger* (Hook.) Benth.]	California	4, 6, 8, 11		
	*Eremocarpus setigerus* (Hook.) Benth.	California			1, 10, 11
	*Euphorbia* sp.	California	3, 6, 9, 11	3	
	*Euphorbia albomarginata* Torrey & A. Gray	California			
	*Euphorbia amygdaloides L.* ssp*. amygdaloides*	Europa		3	
	*Euphorbia antisyphillitica* Zucc.	Mexico and South West USA		4, 6, 8, 9	
	*Euphorbia characias* L. ssp*. characias*	Europe		3	
	*Euphorbia grantii* Oliv.	Mexico, South West USA			3
	*Euphorbia lathyris* L.	Eurasia, Africa		4	
	*Euphorbia ocellata* Durand & Hilg. ssp. *ocellata*	California			
	*Euphorbia peplus* L.	Eurasia, Africa		3	
	*Euphorbia polycarpa* Benth.	California	11		11
	*Euphorbia serrata* L.	Europe, Africa		3	
	*Euphorbia villosa* Waldst. & Kit. ex Willd.	Europe		3	
	*Jatropha cinerea* (Oretga) Mull.	Mexico and South West USA		3, 9	
	*Jatropha curcas* L.	Mexico		4, 7, 9	
	*Jatropha dioica* Sesse	Mexico and Texas		3, 9	
	*Ricinus communis* L.	Africa introduced from Europe		4, 8, 12	
	*Synadenium grantii* Hook.	Asia			3
*Fabaceae*	*Acacia cochliacantha* Bonpl. ex Willd.	Mexico		4, 6, 10, 12	
	*Acacia cornigera* (L.) Willd.	Mexico		3	
	*Acacia farnesiana* (L.) Willd. [*Vachellia farnesiana* (L.) Wight & Arn.]	Mexico		1, 4, 6, 11	
	*Acmispon glaber* (Vogel) Brouillet [*Lotus scoparius* (Nutt. in Torr. & A. Gray) Ottley]	California	10		
	*Acosmium panamense* (Benth.) Yakoviev	Mexico		4, 10, 12	
	*Bauhinia divaricata* L.	Jamaica		4, 6, 10, 12	
	*Caesalpinia pulcherrima* (L.) Sw.	Mexico		10	
	*Calliandra californica* Benth.	California and Mexico		12	
	*Ceratonia siliqua* L.	Mediterranean region		3, 4, 11	
	*Cercis occidentalis* Torrey	California			
	*Crotalaria incana* L.	Mexico		10	
	*Desmodium incanum* DC.	Mexico		3, 4, 6, 10, 12	
	*Enterolobium cyclocarpum* (Jacq.) Griseb.	Mexico		3	
	*Erythrina corallodendron* L.	Mexico			11
	*Eysenhardtia polystachya* (Ortega) Sarg.	Mexico		13	
	*Gliricidia sepium* (Jacq.) Kunth ex Walp	Mexico		6, 12	
	*Glycyrrhiza glabra* L.	Eurasia		4	
	*Haematoxylon brasiletto* H. Karst	Mexico		8, 12, 13	
	*Haematoxylon campechianum* L*.*	Mexico		1, 4, 6	
	*Hoita macrostachya* (DC.) Rydb.	California	3, 11		
	*Hoita orbicularis* (Lindl.) Rydb.	California	1, 11		
	*Indigofera suffruticosa* Mill.	Mexico		3, 4, 6, 11	
	*Inga jinicuil* G. Don	Mexico		1, 4, 6	
	*Lathyrus vestitus* Nutt.	California	4, 11		
	*Lens culinaris* Medic.	Asia		14	
	*Lupinus* sp.	California	11, 13		
	*Lupinus arboreus* Sims	California			
	*Lupinus cytisoides* J. Agardt (*L. latifolia* J. Agardt.)	California			
	*Lysiloma acapulcensis* Benth.	Mexico		3	
	*Medicago sativa* L.	Asia introduced from Europe		1, 8	
	*Mimosa tenuiflora* (Willd.) Poir.	Mexico		3, 4	
	*Mucuna pruriens* (L.) DC.	Africa		4	
	*Ononis spinosa* L.	Eurasia, Africa		4	
	*Olneya tesota* A. Gray	California and Mexico		4, 10, 12	
	*Phaseolus vulgaris* L.	Central America cultivated from all continents		5	
	*Pisum sativum* L.	Mediterranean region		14	
	*Pithecellobium dulce* (Roxb.) Benth.	Mexico		4, 10	
	*Prosopis* sp*.*	California and Mexico		3, 4, 9	
	*Prosopis juliflora* (Sw.) DC.	Mexico		3, 4, 9	
	*Prosopis grandulosa* Torr.	California	3, 9		
	*Stylosanthes viscosa* (L.) Sw.	Mexico			4, 6
	*Tamarindus indica* L.	India			
	*Trifolium* sp.	California	4		
	*Vicia faba* L.	Eurasia		4, 10	
	*Vicia gigantean* Hook.	California	4		
	*Castanea sativa* Mill.	Eurasia		4	
	*Notholithocarpus densiflorus* (Hook. & Arn.) Manos, C. H. Cannon, & S. Oh [*Lithocarpus densiflorus* (Hook. & Arn.) Rehd.]	California	3, 11		
	*Quercus* sp.	California, Mexico	1, 3, 9	1, 9, 11	
	*Quercus agrifolia* Nee	California	1, 3, 4		3, 4, 10
	*Quercus dumosa* Nutt.	California	3, 9, 11		
	*Quercus faginea* Lam.	Mediterranean region		3	
	*Quercus ilex* L.	Europe		1, 3, 4, 14	3, 4, 10
	*Quercus ilex* ssp. *ballota* (Desf.) Samp.	Mediterranean region		2, 3	
	*Quercus oleoides* Schltdl. & Cham.	Mexico		9	
	*Quercus lobata* Nee	California	4		
	*Quercus robur* L.	Eurasia		4	
	*Quercus turbinella* Greene	California	3, 9		
*Frankeniaceae*	*Frankenia salina* (Molina) I. M. Johnst. (*F. grandifolia* Cham. & Schltdl.)	California	4		
*Gentianaceae*	*Centaurium erythraea* Raf.	Europe		1, 4, 8, 11	1, 10, 11
	*Centaurium venustum* (A. Gray) B. L. Rob. [*Zeltnera venusta* (Gray) G.Mans.]	California	1, 11		1, 11
	*Zeltnera venusta* (A. Gray) Mansion (*Erythraea venusta* A. Gray)	California	1, 6, 11		
*Geraniaceae*	*Geranium lucidum* L.	Eurasia, Africa		3	
	*Geranium robertianum* L.	Europe		4	
	*Pelargonium* sp.	South Africa, Introduced from Europe		4	
*Gesneriaceae*	*Konleria deppeana* (Schltdl. & Cham.) Fritsch	Mexico		4, 13	
*Grossulariaceae*	*Ribes indecorum* Eastw.	California	9		
*Hypericaceae*	*Hypericum androsaemum* L.	Eurasia		3	
	*Hypericum balearicum* L.	Spain's Balearic Islands		5	
	*Hypericum perforatum* L.	Eurasia		3, 4, 7, 8, 12	
*Illiciaceae*	*Illicium verum* Hook.f.	Asia		4	
*Iridaceae*	*Crocus sativus* L.	Europe			8
	*Iris* sp.	California	4		
	*Iris douglasiana* Herbert	California			
	*Sisyrinchum bellum* S. Watson	California	4, 5, 7, 11		10, 11
*Juglandaceae*	*Juglans californica* S. Wats.	California	1		
	*Juglans regia* L.	Balkan Peninsula, Asia		1, 2, 3, 4, 5, 6, 7, 10, 11	
*Juncaceae*	*Juncus* sp. (mainly, *J. effusus* L.; *J. inflexus* L., and *J. conglomeratus* L.)	Eurasia, Africa		3	
	*Juncus textilis* Buchenau	California	3		
*Krameriaceae*	*Krameria grayi* Rose & Painter (*K. bicolor* S. Watson)	California		3, 4, 12	
*Lamiaceae*	*Agastache mexicana* (Kunth) Lint & Epling	Mexico		8	
	*Calamintha nepeta* (L.) Savi	Europe, Africa		8	
	*Clinopodium douglasii* (Benth.) Kuntze [*Micromeria douglasii* (Benth.) Kuntze; *Satureja douglasii* (Benth.) Briq.]	California	3, 4, 5, 8, 9, 10, 11		4
	*Dracocephalum moldavica* L.	Asia, introduced from Europe		1	
	*Hyptis mutabilis* (Rich.) Briq.	Mexico		4	
	*Hyptis stellulata* Benth.	Mexico		3, 4, 7, 8, 9	
	*Hyptis verticillata* Jacq*.*	Mexico		3, 4	
	*Hyptis emoryi* Torrey [*Condea emoryi* (Torr.) Harely & J. F. B. Pastore]	California	1		1
	*Lavandula angustifolia* Mill. (*L. vera* DC.; *L. spica* L.)	Europe			
	*Lavandula latifolia* Medik.	Mediterranean region		3, 8, 14	
	*Lavandula spica* L.	Mediterranean region			4
	*Leonurus japonicus* Hoult	Asia		5, 6, 10	
	*Lepechinia calycina* (Benth.) Epling	California			7, 10, 11
	*Lepechinia caulescens* (Ortega) Epling	Mexico		4, 6	
	*Marrubium vulgare* L.	Europe		3, 4, 11	3, 6, 8, 10
	*Melissa officinalis* L.	Europe		1, 4, 8, 11	1
	*Mentha* sp.	California	3, 4, 7, 8, 9, 13		
	*Mentha arvensis* L.	Eurasia, California	9		13
	*Mentha longifolia* (L.) Huds.	Eurasia, Africa		4	
	*Mentha pulegium* L.	Eurasia, Africa introduced from America		4	1
	*Mentha spicata* L.	Europe introduced from California		4, 8	10
	*Mentha suaveolens* Ehrh.	Mediterranean region		3, 4, 10	
	*Mentha x gentilis* L.	Europe		8	
	*Mentha* x *piperita* L. (*M. aquatica* L. x *M. spicata* L.)	Europe introduced from California		4, 8	10
	*Monardella villosa* Benth.	California	1, 4, 10		
	*Ocimum basilicum* L.	Africa		8	
	*Origanum majorana* L.	Europe		6, 7	
	*Origanum vulgare* L. ssp. *vulgare*	Europe		4, 8, 11	
	*Phlomis lychnitis* L.	Europe		4	
	*Rosmarinus officinalis* L.	Europe		1, 3, 4, 5, 6, 7, 8, 11, 14	3, 4, 9, 10, 11
	*Salvia* sp.	California, Mexico	8, 11	3, 5	3, 8
	*Salvia aethiopis* L.	Europe			3, 8
	*Salvia apiana* Jepson [*Ramona polystachya* (Benth.) Greene]	California	4, 8, 9, 10		
	*Salvia carduaceae* Benth.	California	5		
	*Salvia columbariae* Benth.	California	1, 3, 4, 6, 9, 11		
	*Salvia lavandulifolia* Vahl	Europe		8	
	*Salvia lavanduloides* Kunth	Mexico		10	
	*Salvia leucantha* Cav.	Mexico		7, 12	
	*Salvia mellifera* E. Greene [*Ramona stachyoides* (Benth.) Briq.]	California	1, 4, 8, 9, 10		3
	*Salvia officinalis* L.	Europe		1, 3, 8, 10, 14	
	*Salvia spathacea* E. Greene	California	1, 7, 11		
	*Salvia verbenaca* L.	Eurasia, Africa		2, 4, 5, 11	
	*Satureja douglasii* (Benth.) Briq. [*Clinopodium douglasii* (Benth.) Kuntze]	California	1, 3, 4, 5, 7, 8, 9, 10, 11, 13		3, 4, 7, 8
	*Satureja hortensis* L.	Eurasia		3, 10	
	*Satureja macrostema* (Moc. & Sesse ex Benth.) Briq.	Mexico		4, 6	
	*Stachys albens* A. Gray	California	3, 4, 10		
	*Stachys bullata* Benth	California	3, 9, 10		
	*Teucrium chamaedrys* L.	Mediterranean region		9	
	*Teucrium scorodonia* L.	Europe, Africa		3	
	*Thymus* sp.	Eurasia, Africa		7, 11	
	*Thymus mastichina* (L.) L.	Spain		11	
	*Thymus praecox* Opiz	Europe		8	
	*Thymus vulgaris* L.	Mediterranean region		1, 2, 3, 4, 6, 7, 8, 11, 12	
	*Thymus zygis* L.	Spain, Africa		11	
	*Trichostema lanatum* Benth.	California	3, 4, 5, 6, 7, 8, 11		
	*Trichostema lanceolatum* Benth.	California	3, 4, 6, 7, 9, 10, 11, 13		3, 6, 8, 9, 10
*Laminariaceae*	*Laminaria* sp.	California	4		
	*Macrocystis* sp.	California	4		
*Lauraceae*	*Laurus nobilis* L.	Europe		3, 4, 7, 11	
	*Umbellularia californica* (Hook. & Arn.) Nutt.	California	3, 4, 7, 8, 10		8
*Liliaceae*	*Lilium candidum* L.	Balkan Peninsula, Middle East		3	
	*Prosartes parvifolia* S. Watson [*Disporum hookeri* (Torr.) G. Nicholson]	California	13		
	*Hesperolinon californicum* (Benth.) Small	California	11		
	*Linum usitatissimum* L.	Asia, Africa		11	
*Loasaceae*	*Mentzelia* sp.	California	11		
	*Mentzelia aspera* L.	California and Mexico		6	
	*Mentzelia hispida* Willd.	California		4, 6	
*Lythraceae*	*Cuphea aequipetala* Cav.	Mexico		3, 4	
	*Heimia salicifolia* Link.	Mexico		3, 12	
*Magnoliaceae*	*Magnolia grandiflora* L.	Mexico and South West USA		1, 8	
*Malpighiaceae*	*Byrsonima crassifolia* (L.) Kunth.	Mexico		3, 4	
	*Galphimia glauca* Cav.	Mexico		3, 7	
*Malvaceae*	*Abutilon palmeri* A. Gray	California			1, 3, 11
	*Alcea rosea* L.	China introduced from Europe		8, 11	
	*Althaea officinalis* L.	Eurasia, Africa		4, 11	
	*Ceiba pentandra* (L.) Gaertn.	Mexico		3, 4, 8	
	*Chiranthodendron pentadactylon* Larreategui	Mexico		1, 8	
	*Fremontodendron californicum* (Torrey) Cov.	California	10		
	*Guazuma tomentosa* Kunth (*G. ulmifolia* Lam.)	Mexico		3, 4, 6, 12	
	*Hibiscus sabdariffa* L.	West of Africa		4, 8, 12, 13	
	*Hibiscus rosa-sinensis* L.	West of Africa		9, 12	
	*Malacothamnus* sp. *(Malvastrum* sp.)	California	4, 5, 7		
	*Malacothamnus fasciculatus* (Torrey & A. Gray) E. Greene	California	4		
	*Malva moschata* L.	Eurasia		11	
	*Malva neglecta* Wallr.	Eurasia		3, 4, 11, 14	
	*Malva parviflora* L.	Europe		3, 4	
	*Malva sylvestris* L.	Europe		1, 3, 4, 6, 5, 7, 8, 9, 10, 11, 14	1, 3, 11
	*Malvaviscus arboreus* Cav*.*	Mexico		4, 6	
	*Pavonia schiedeana* Steud.	Mexico		1, 3, 4, 6	
	*Pseudobombax ellipticum* (Kunth) Dugard	Mexico		10	
	*Sida acuta* Burm.	Mexico		3, 4, 13	
	*Sida rhombifolia* L.	Mexico		7, 8	
	*Sphaeralcea emoryi* Torr. ex A. Gray	California			3, 9
	*Theobroma cacao* L.	Mexico, Amazon basin		11	
	*Tilia* sp.	Eurasia, Mexico		8	
	*Tilia cordata* Mill.	Europe		8	
	*Tilia platyphyllos* Scop. ssp. *platyphyllos*	Eurasia		4, 8	
*Martyniaceae*	*Martynia annua* L.	Mexico		8	
*Melanthiaceae*	*Trillium chloropetalum* (Torrey) Howell	California	1, 4		
	*Zigadenus fremontii* (Torr.) S. Watson [*Toxicoscordion fremontii* (Torr.) Rydb.]	California	3		
	*Zigadenus venenosus* (S. Watson) Rydb. [*Toxicoxcordion venenosus* (S. Watson) Rydb.]	California	3		
*Melastomataceae*	*Miconia albicans* (Sw.) DC.	Mexico		4, 13	
*Meliaceae*	*Cedrela odorata* L.	Mexico		4, 9, 12	
*Menispermaceae*	*Cissampelos pareira* L.	Africa		1, 4	
*Montiaceae*	*Claytonia perfoliata* Willd.	California	4		
*Moraceae*	*Brosimum alicastrum* Sw.	Mexico		3, 10, 13	
	*Dorstenia contrajerva* L*.*	Mexico		7, 12	
	*Ficus carica* L.	Middle east, western Asia		3, 11	
	*Ficus petiolaris* Kunth	Mexico		1, 4, 7	
*Muntingiaceae*	*Muntingia calabura* L.	Mexico		3, 6, 7	
*Musaceae*	*Musa* sp.	Asia		11	
	*Musa sapientum* L. (*Musa* x *paradisiac* L.)	Indonesian, grown in countries with tropical climate		4	3, 11
*Myrtaceae*	*Eucalyptus* sp.	Australia			8
	*Eucalyptus globulus* Labill.	Australia		3, 11	
	*Eugenia acapulcensis* Steud.	Central America		4, 6, 10	
	*Myrtus communis* L.	Europe			
	*Psidium guava* L.	Central America and Mexico		4	
*Nyctaginaceae*	*Abronia* sp.	California	13		
*Oleaceae*	*Forestierra pubescence* Nutt. (*F*. *neomexicana* A. Gray)	California			8
	*Fraxinus angustifolia Vahl* ssp*. angustifolia*	Europe		2	
	*Fraxinus dipetala* Hook. & Arn.	California	3, 11		
	*Fraxinus excelsior* L.			1, 3	
	*Fraxinus latifolia* Benth.	California	11		11
	*Fraxinus uhdei* (Wenz.) Lingel.	Mexico		12	
	*Jasminum officinale* L.	Middle East, India, China			4, 6, 8
	*Ligustrum parteri* Coult. & Rose	Europe		1, 3	
	*Olea europaea* L. var. *europaea*	Mediterranea region		1, 3, 4, 13	
	*Olea europaea* L. var. *sylvestris* (Mill.) Brot.	Mediterranea region		1	
*Onagraceae*	*Ludwigia octovalvis (Jacq.) P. H. Ravens*	Central America		3	
	*Epilobium canum* (E. Greene) Raven (*Zauschneria californica* C. Presl)	California	3, 7, 11, 13		
	*Gaura coccinea* Nutt. Ex Pursh [*Oenothera suffrutescens* (Ser.) W. L. Wagner & Hoch]	California			1, 4
	*Oenothera albicaulis* Pursh	Mexico, West USA			11
	*Oenothera elata* Kuth	California			
	*Oenothera hookeri* Torrey & A. Gray	California			
	*Oenothera rosea* L’Her. Ex Aiton	Mexico and Texas		3, 4	
*Orobanchaceae*	*Castilleja* sp.	California	3		
	*Castilleja affinis* Hook. & Arn.	California	3		
	*Castilleja attenuata* (A. Gray) Chuang & Heckard	California	10		
	*Castilleja elastica* Sesse ex Cerv.	Mexico		7, 12	
	*Castilleja tenuiflora* Benth.	Mexico and South West USA		1, 3, 7, 12	
	*Orthocarpus* sp.	California	10		
*Paeoniaceae*	*Paeonia brownii* Hook.	California	1, 4, 10		
	*Paeonia californica* Torrey & A. Gray	California	1, 3, 4, 5, 7, 8, 10, 11, 13		3, 4, 8, 10
*Papaveraceae*	*Argemone mexicana* L. (*A. sanguinea Greene*)	Mexico		3, 7, 9, 12, 13	
	*Chelidonium majus* L.	Eurasia		1, 3, 8, 11	
	*Eschscholzia* sp.	California	8		
	*Eschscholzia californica* Cham.	California	3, 4, 8, 9		
	*Fumaria officinalis* L. ssp. *officinalis*	Eurasia, Africa		3	
	*Papaver rhoeas* L.	Eurasia, Africa		4, 8, 11	
	*Papaver somniferum* L.	Eastern Mediterranean, introduced from Eurasia		8	
	*Romneya coulteri* Harv.	California	3, 4, 9		
*Pelliaceae*	*Pellia californica* Cham.	California	11		
*Petiveraceae*	*Petiveria alliacea* L.	Mexico		3, 8, 12	
	*Rivina humilis* L.	Mexico		3, 4, 12	
*Phrymaceae*	*Mimulus aurantiacus* Curtis (*M. puniceus* Nutt.)	California	3, 5, 13		
	*Mimulus glutinosus* J. C. Wendl. (*M. aurantiacus* Torr.)	California			
	*Mimulus guttatus* DC	California	4		
*Picrodendraceae*	*Petalostigma pubescens* Domin	Australia, New Guinea			10, 11
*Pinaceae*	*Abies concolor* (Gordon & Glend.) Lindley	California			4, 6
	*Pinus* sp.	California	1, 3, 7, 8, 9, 10, 11	1, 3, 4, 11	7, 8
	*Pinus halepensis* Mill.	Mediterranean region		3, 11, 12	
	*Pinus monophylla* Torrey & Fremont	California	10, 11		
	*Pinus patula* Schiede ex Schltdl. & Cham.	Mexico		10	
	*Pinus pinaster* Aiton.	Europe		3	
	*Pinus sabiniana* Douglas	California	3, 7		
	*Pinus sylvestris* L.	Eurasia		11	
	*Pseudotsuga menziesii* (Mirb.) Franco	California			6, 11, 13
*Piperaceae*	*Peperomia pellucida* Kunth	South and Central America		3, 4	
	*Piper sanctum* (Miq.) Schltdl. Ex C. DC.	Mexico		8	
*Plantaginaceae*	*Antirrhinum nuttallianum* Benth.	California	10		
	*Digitalis minor* L.	Spain’s Balearic Islands		1	
	*Digitalis purpurea* L.	Europe			
	*Globularia alypum* L.	Mediterranean region		1	
	*Keckiella antirrhinoides* (Benth.) Straw	California			
	*Keckiella breviflora* (Lindley) Straw	California	3, 10		
	*Keckiella cordifolia* (Benth.) Straw (*Penstemon cordifolius* Benth.)	California	3, 10		
	*Penstemon centranthifolius* Benth.	California	3		
	*Plantago* sp.	California	1, 3, 9, 10, 11		
	*Plantago lagopus* L.	Eurasia, Africa		5, 11, 12	
	*Plantago lanceolata* L.	Eurasia		3, 4, 5, 7, 11, 12, 14	
	*Plantago major* L.	Eurasia		1, 3, 7, 11, 12, 14	4, 9, 11
*Platanaceae*	*Platanus lindeliana* Mart. & Gal.	Mexico		7, 8, 10	
	*Platanus racemosa* Nutt.	California	10, 11		
	*Platanus x hispanica* Mill. ex Münch.	Europe		1	
*Plumbaginaceae*	*Limonium californicum* (Boiss.) A. A. Heller	California	1, 6, 10, 11		
	*Plumbago pulchella Boiss.*	Mexico		3, 6, 12	
*Poaceae*	*Arundo donax* L.	Mediterranean region, Asia		2, 10	
	*Avena sativa* L.	Europe and naturalized in California		4	
	*Bouteloua eriopoda* (Torrey) Torrey	California			
	*Coix lachrymal-jobi* L.	Asia		1	
	*Cynodon dactylon* (L.) Pers.	Africa		4	
	*Distichlis spicata* (L.) E. Greene	California	1, 3, 4, 6, 8, 10		
	*Elymus condensatus* (J. Presl) A. Love (*Leymus condensatus* J. Presl)	California	4, 6, 11		
	*Elymus repens* (L.) Gould	Europe			6, 13
	*Oryza* sp*.*	Asia and Europe			3, 4
	*Oryza sativa* L.	Africa, Asia, introduced from all continents		4	3, 4
	*Triticum aestivum* L.	Europe		3, 4, 5, 7, 9, 11, 14	
	*Zea mays* L.	Mexico		1, 2, 3, 4, 11, 12	3, 11
*Polemoniaceae*	*Loeselia mexicana* (Lam.) Brand	Mexico		3	1, 4
	*Navarretia atractyloides* (Benth.) Hook. & Arn.	California	3		
*Polygonaceae*	*Chorizanthe* sp.	California	3, 11		
	*Eriogonum* sp	California	4, 5, 8, 9		
	*Eriogonum elongatum* Benth.	California	1, 10, 11		
	*Eriogonum fasciculatum* Benth.	California	4, 5, 7, 8, 9		3, 6, 8, 10
	*Eriogonum nudum* Benth. [*E. latifolium* Smith ssp. *nudum* (Douglas ex Bentham) S. Stokes]	California	1, 3, 10, 11		10
	*Rheum rhabarbarum* L.	Asia, introduced to California from Europe			7
	*Rumex* sp.	California			
	*Rumex crispus* L.	Eurasia			4, 6, 11
	*Rumex hymenosepalus* Torrey	California	3, 4, 7, 8, 10, 11		4, 8, 10
	*Rumex obtusifolius* L.	Europe		1, 3, 4	
*Polypodiaceae*	*Phlebodium aureum* (L.) J. Sm.	South and Central America		4, 13	
	*Polypodium californicum* Kaulf.	California	1, 3, 7, 11		
*Portulacaceae*	*Portulaca oleracea* L.	Eurasia, introduced to Mexico		1	
*Primulaceae*	*Anagallis arvensis* L. [*Lysimachia arvensis* (L.) U. Manns & Anderb.]	Europe	3, 6	1, 3, 6, 11	
	*Anagallis foemina* Mill. (*Lysimachia foemina* Mill.)	Europe		6	
	*Primula elatior* L. ssp. *elatior*	Europe		7	
	*Primula veris* L.	Eurasia		3	
*Pteridaceae*	*Adiantum aleuticum* (Rupr.) C.A. Paris (*A. pedatum* L.)	California, Europe, Mexico	1, 4, 7		
	*Adiantum capillus-veneris* L.	California		3, 4, 5, 10, 11, 13	1, 3, 4, 7
	*Adiantum jordanii* Mueller	California	1, 4, 5, 7, 8, 11		1, 4
	*Pellaea andromedifolia* (Kaulf.) Fee	California	1, 4, 5, 7		
	*Pellaea atropurpurea* (L.) Link	Mexico			3, 11, 13
	*Pellaea mucronata* (D. Eaton) D. Eaton (*P. ornithopus* Hook.)	California	1, 3, 4, 11		
	*Pentagramma triangularis* (Kaulf.) G. Yatskievych, Windhan & Wollenweber	California	3, 7		
*Ranunculaceae*	*Actaea rubra* (Aiton) Willd.	California			11
	*Aquilegia* sp.	California			4
	*Aquilegia truncate* Fisch. ex DC.	California			
	*Clematis lasiantha* Nutt.	California	3, 6		
	*Clematis ligusticifolia* Nutt.	California	1, 3, 6, 10		1, 3, 10, 11
	*Clematis pauciflora* Nutt.	California	3, 10, 11, 13		
	*Clematis virginiana* L.	Eastern U.S.A.			
	*Helleborus viridis* L. ssp. *occidentalis* (Reut.) Schiffn.	Europe		4	
	*Ranunculus* sp.	California	3		
	*Ranunculus ficaria* L.	Eurasia		3	
*Resedaceae*	*Reseda alba* L.	Eurasia, Africa		4	
*Rhamnaceae*	*Ceanothus* sp.	California	3		
	*Ceanothus arboreus* Greene	California			
	*Ceanothus leucodermis*Greene	California			7
	*Ceanothus thyrsiflorus* Eschsch.	California			
	*Ceanothus verrucosus* Nutt.	California			
	*Frangula californica* (Eschsch.) A. Gray ssp. *occidentalis* (*Rhamnus californica* Eschsch.)	California	1, 3, 4, 6, 7, 11		
	*Frangula purshiana* (DC.) Cooper (*Rhamnus purshiana* DC.)	California	4		
	*Gouania polygama* (Jacq.) Urb.	Mexico		6, 8, 10	
	*Karwinskia humboldtiana* (Schult.) Zucc. (*Rhamnus humboldtiana Schult.*)	Mexico and Texas		8	
	*Rhamnus alaternus* L.	Mediterranean region		1, 11	
	*Rhamnus californica* Eschsch. [*Frangula californica* (Eschsch.) A. Gray]	California	3, 4, 7		4
	*Rhamnus crocea* Nutt.	California			
	*Rhamnus ilicifolia* Kellogg	California	6, 10		
*Rhizophoraceae*	*Rhizophora mangle* L.	Mexico		1, 6, 13	
*Rhodomelaceae*	*Alsidium helminthochorton* (Schw.) Kütz.	Not documented		4	
*Rosaceae*	*Adenostoma* sp.	California	3, 4, 7, 8		
	*Adenostoma fasciculatum* Hook. & Arn.	California	3, 5, 7, 11		
	*Adenostoma sparsifolium* Torr.	California	1, 3, 4, 6, 8, 9, 10, 11		
	*Agrimonia eupatoria* L. ssp. *euptoria*	Europe		4, 11	
	*Chamaebatia foliolosa* Benth.	California			3, 4
	*Cercocarpus betuloides* Torrey & A. Gray	California	4, 10		
	*Crataegus monogyna* Jacq.	Eurasia, Africa		1, 3, 4, 8, 11, 14	
	*Cydonia oblonga* Mill.	Asia introduced to California from Europe		4, 8, 9	
	*Eriobotrya japonica* (Thunb.) Lindl.	Asia		4	
	*Heteromeles arbutifolia* (Lindley) Roemer (*Photinia arbutifolia* Lindl.)	California	3, 5		
	*Horkelia cuneata* Lindl.	California	1, 4, 6, 8, 10, 11		
	*Malus domestica* (Borkh.) Borkh.	Asia introduced from Europe, North America		1, 3, 4, 8, 11	
	*Malus sylvestris* Mill.	Europe		4	
	*Potentilla glandulosa* Lindl. [*Drymocallis glandulosa* (Lindl.) Rydb.]	California	1, 4, 6, 8, 10, 11		
	*Potentilla reptans* L.	Eurasia, Africa		1, 3, 13	
	*Prunus avium* (L.) L.	Eurasia		2, 4, 12	
	*Prunus cerasus* L.	Eurasia		4, 10	
	*Prunus domestica* L.	Asia		4	
	*Prunus dulcis* (Mill.) D.A. Webb	Asia		11	
	*Prunus emarginata* (Hook.) Walp.	California	3, 7, 11		
	*Prunus ilicifolia* (Nutt. Ex Hook. & Arn.) Walp. [*Cerasus ilicifolia* Nutt. Ex Hook & Arn.]	California	4, 10		
	*Prunus integrifolia* (C. Presl) Walp.	South America			
	*Prunus serotina* Ehrh.	Mexico, South West USA		10	
	*Prunus spinosa* L.	Eurasia		1, 3, 4, 10, 11	
	*Prunus virginiana* L. var. *demissa* (Nutt.) Torrey [*Cerasus virginana* (L.) Michx.]	California	4, 10		
	*Rosa* sp.	Eurasia		9	
	*Rosa agrestis* Savi	Europe		4, 14	
	*Rosa californica* Cham. & Schldl.	California	3, 4, 7, 9, 10, 11, 13		9
	*Rosa canina* L.	Eurasia, Africa		4, 11	
	*Rosa gallica* L.	Eurasia			4, 9
	*Rubus ulmifolius* Schott	Europe, Africa, introduced from California		1, 3, 4, 5, 7, 11	
	*Rubus ursinus* Cham. & Schldl (*R. vitifolius* Cham. & Schldl.)	California	3, 4, 5, 6		4
	*Sorbus domestica* L.	Eurasia, Africa		4	
*Rubiaceae*	*Cinchona officinalis* L.	South America			10
	*Coffea arabica* L.	Africa		4, 7, 8, 11	
	*Galium angustifolium* Nutt.	California	4		
	*Galium triflorum* Michaux	California	3, 4, 7		
	*Hamelia patens* Jacq.	Mexico		3, 12	
*Rutaceae*	*Amyris madrensis* S. Watson	Mexico		3	
	*Amyris texana* (Buckley) P. Wilson	Mexico		3, 7	
	*Casimiroa edulis* La Llave & Lex.	Mexico		1, 8	
	*Citrus* sp.	Australia, introduced from Europe			
	*Citrus limon* (L.) Burm fil. (pro. sp.)	Asia, introduced from Europe		1, 3, 4, 5, 6, 8, 11	7, 11
	*Citrus sinensis* L. Osbeck	Asia, introduced from Europe		4, 8, 11, 14	7, 8, 10
	*Ruta chalepensis* L.	Eurasia, Africa		1, 4, 10	
	*Ruta graveolens* L.	Europe			8, 9
*Salicaceae*	*Populus balsamifera* L. ssp. *trichocarpa* (Torrey & A. Gray) Brayshaw (*P. trichocarpa* Hook.)	California	3, 7		3
	*Populus fremontii* S. Watson	California	3, 7, 8		3, 11
	*Populus tremuloides* Michaux	California			3
	*Salix* sp.	California	3, 10		
	*Salix exigua* Nutt.	California	3, 8		
	*Salix laevigata* Bebb	California	4, 11		
	*Salix lasiolepis* Benth	California	1, 6, 8, 10, 11		
*Salviniaceae*	*Salvinia minima* Baker	Mexico			4
*Santalaceae*	*Arceuthobium* sp.	California			3
	*Phoradendron californicum* Nutt.	California		3, 4, 6	
	*Phoradendron juniperinum* Engelm. Ex A. Gray	California	3, 9		
	*Phoradendron macrophyllum* (Engelm.) Cockerell	California	5, 7, 9		
	*Phoradendron serotinum* (Raf.) M. C. Johnst. spp. *macrophyllum*(Engelm.) Kuijt	California	5, 7		
	*Phoradendron serotinum* (Raf.) M. C. Johnst. ssp. *tomentosum* (DC.) Kuijt [*P. leucarpum* (Raf.) Reveal & M. C. Johnst. ssp. *tomentosum* (DC.) J. R. Abbott & R. L. Thomps.; *P. coloradensa* Raf.]	California	3, 7, 11		
	*Phoradendron villosum* Nutt.	California	3, 5, 7, 9		
	*Phoradendron villosum* Nutt. [*P. flavescens* (Pursh.) Nutt.]	California	5, 7, 9		
	*Viscum album* L. ssp. *album*	Eurasia		1, 10	
*Sapindaceae*	*Aesculus californica* (Spach) Nutt.	California	1, 3, 8, 9		1
	*Aesculus hippocastanum* L.	Europe		1, 7, 13	
	*Dodonaea viscosa* Jacq.	Mexico		3, 4, 6, 7, 12	
*Sapotaceae*	*Achras zapota* L. [*Manilkara zapota* (L.) P. Royen]	Mexico		4, 7, 8, 9, 12, 13	
	*Manilkara* sp.	Mexico			3
*Sarraceniaceae*	*Darlingtonia californica* Torr.	California			
*Saururaceae*	*Anemopsis californica* (Nutt.) Hook. & Arn.	California, Mexico	1, 2, 3, 6, 7, 8, 10, 11, 13	3, 5, 7, 11	1, 3, 7, 10
*Scrophulariaceae*	*Buddleja americana* L.	Mexico		13	
	*Capraria biflora* L.	Mexico		10	
	*Russelia sarmentosa Jacq.*	Europe		3, 4, 8	
	*Scrophularia alpestris* Gay ex Benth.	Europe		3	
	*Scrophularia balbisii* Hornem. ssp. *balbisii*	Eurasia, North America		3, 7	
	*Scrophularia californica* Cham. & Schldl.	California	3, 6, 9, 11		
	*Verbascum sinuatum* L.	Eurasia, Africa		1, 3, 11, 13	
	*Verbascum thapsus* L.	Europe		9, 11	4
*Selaginellaceae*	*Selaginella lepidophylla* (Hook. & Grev.) Spring	Mexico		12, 13	
*Simaroubaceae*	*Castela texana* (Torr. & A. Gray) Rose	Mexico and Texas		4	
	*Castela tortuosa* Liebm.	Mexico		6	
*Simmondsiaceae*	Simmondsia *chinensia* (Link) C.K.Schneid.	California, Mexico			3, 7, 11
*Smilacaceae*	*Smilax ornata* Lem.	Mexico			1, 10
	*Smilax lanceolata* L.	Southeastern USA		1, 3, 4, 7, 10, 12	
*Solanaceae*	*Atropa belladonna* L.	Europe, naturalized in California			
	*Capsium annuum* L.	Mexico			
	*Datura innoxia* Mill.	California			10
	*Datura stramonium* L.	Mexico introduced from Europe		3, 7, 10, 11, 12	
	*Datura wrightii* Regel	California	1, 3, 4, 7, 8, 9, 10, 11		
	*Hyoscyamus albus* L.	Eurasia		4, 8, 11	
	*Nicotiana* sp.	California	3, 4, 5, 6, 7, 9, 10, 11		
	*Nicotiana attenuata* Torrey	California	3, 4, 6		
	*Nicotiana clevelandii* A. Gray	California	11		
	*Nicotiana glauca* Graham	South America		7, 8, 10, 12	
	*Nicotiana quadrivalis* Pursh (*N. bigelovii* Torr.)	California	3, 4, 8, 9, 10, 11		
	*Nicotiana pusilla* Blanco. *(N. rustica* L*.)*	Mexico		7, 8, 10, 12	
	*Nicotiana tabacum* L.	Mexico			4, 8, 10
	*Solanum* sp.	California			3
	*Solanum carolinense* L.	USA			
	*Solanum douglasii* Dunal	California	3, 9, 11		
	*Solanum lycopersicum* L.	Central and South America		3	
	*Solanum melongena* L.	Asia		4	
	*Solanum nigrum* L.	California	3, 6, 9		
	*Solanum tuberosum* L.	South America		3, 7	3
*Sterculiaceae*	*Waltheria americana L.*	*Mexico*		3, 10, 12	
*Tropaeolaceae*	*Tropaeolum majus* L.	California			11
*Turneraceae*	*Turnera diffusa* Willd. ex Schult.	Southern Texas, Mexico, South America, Caribbean		7, 8, 10, 11	7, 10
*Typhaceae*	*Typha latifolia* L.	California	1		
*Urticaceae*	*Cecropia obtusifolia* Bertol.	Mexico		4, 13	
	*Parietaria judaica* L.	Eurasia, Africa		1, 3, 4, 12	
	*Urtica* sp.	California	1, 3, 5, 7, 8		
	*Urtica dioica* L.	Eurasia, Africa		1, 2, 3, 4, 5, 6, 7, 8, 11, 14	
	*Urtica dioica* L. ssp. *holosericea* (Nutt.) Thorne	California	7, 8, 10, 11		4, 6
	*Urtica urens* L.	Eurasia		1, 3, 7	
*Ustilaginaceae*	*Ustilago maydis* (Persoon) Roussel	Mexico		7	
*Verbenaceae*	*Aloysia citrodora* Palau	South America introduced from Europe		4, 8	1, 10
	*Aloysia triphylla* (L’Her.) Britton	South America introduced from Europe		4, 8	
	*Verbena bipinnatifida* Nutt. [*Glandularia bipinnatifida* (Nutt.) Nutt.]	Mexico			10
	*Verbena lasiostachys* Link var. *lasiostachys*	California	3, 4, 6, 11		
	*Verbena officinalis* L.	Europe		1, 3, 4, 6, 7, 11, 14	
*Violaceae*	*Viola* sp.	California	3		6
	*Viola riviniana* Rchb.	Europe		1	
*Vitaceae*	*Vitis* sp.	California	1		
	*Vitis vinifera* L.	Europe		1, 3, 4, 7, 10, 11	7, 10
*Zosteraceae*	*Phyllospadix torreyi* S. Wats.	California	11		
*Zygophyllaceae*	*Guaiacum officinale* L.	Caribbean			3, 10
	*Guaiacum sanctum* L.	Mexico		4, 6, 10	
	*Kallstroemia grandiflora* A. Gray	Mexico and South West USA		3, 7, 12	
	*Larrea tridentata* (DC.) Cov. (*L. californica* DC.; *L. mexicana* Moric)	California, Mexico	1, 3, 4, 6, 7, 8, 10, 11	3, 4, 5, 6, 7, 11, 13	

*Botanical family classification and nomenclature for species names were authenticated according to Hickman [45], Stevens [46], and the International Plant Names Index (www.ipni.org)

We assumed if information concerning California medicinal plants was shared by the Native Americans with the Spanish priests some of these species would have been subsequently introduced to Spain as had medicinal plants from Mexico and South America.

Twelve of 265 taxa used by Native Americans were also used in Mexico: *Adiantum aleuticum* (Rupr.) C.A. Paris, *Anemopsis californica* (Nutt.) Hook. & Arn, *Artemisia ludoviciana* Nutt, *Baccharis glutinosa* Pers., *Cucurbita foetidissima* Kunth, *Equisetum arvense* L., *Larrea tridentata* (DC.) Cov., *Opuntia* sp., *Quercus* sp., *Rorippa nasturtium-aquaticum* (L.) Hayek, *Salvia* sp. and *Sambucus mexicana* C. Presl. (Table 3). It is important to point out that these medicinal plants were not necessarily used to treat the same ailments. It is evident that many of the Mission priests and early Spanish explorers were open to the use of Native American medicinal plants and adopted them when medicinal supplies from Spain and Mexico were not available [26, 58–61].

The close reading of diaries, journals, reports, and books indicate there are reasons to believe that sharing of information about medicinal plants did take place at the Missions, but conditions at the Missions and other factors also interfered with the exchange. Table 4 summarizes references that report on the sharing of information. The primary support comes from diaries and reports of priests and others present during the Mission period who observed the use of plants native to California and the introduction of European species period (see Table 4). Direct evidence of the sharing of information comes from reports that neophytes were sent out to collect both food and medicinal plant in times of shortages [64]. A survey conducted in 1812 asked the priests at each Mission to report on the customs and conditions of indigenous people living at or near the Missions [23]. Question no. 15 of the survey asked specifically about the medicinal practices of the people and their use of plants in the treatment of illness. In response to this question, the priest at 13 of the 18 missions reported that the local Native Americans used plants for medicinal purposes. Reports from the other five missions stated that no plants were used by the Native Americans for medicinal purposes. Plant species were identified, ranging in number from one to 14, at eight of the 13 missions reporting the use of medicinal herbs. A total of 16 different plants were reported from all the California Missions.

**Table 4:** Published sources supporting the exchange of information on medicinal plants

1. Reports of an exchange of information	
Comments	Source
Native American teach priests about their medicinal plants (pp. 73-74) (example of exchange of information between Native Americans and priests)	Anderson [26]
Compilation of medicinal plants by Father Garriga (pp. 443-445) (example of exchange of information between Californios and priests)	Beebe and Senkewicz [43]
Father Crespi reports vineyard-like plantings by Native Americans (pp. 45) (example of exchange of information between Native Americans and priests)	Blackburn and Anderson [62]
Sick sailors taken ashore in hope that medicinal herbs could be found (pp. 143) (example of the use of medicinal plant by Spanish explorers in California) Dr. Prat searches for medicinal herbs after first ship land in San Diego (pp. 144) (example of the use of medicinal plant by Spanish explorers in California) List of California plants identified by Portola (pp. 209-293) (example of interest in plants by Spanish explorers)	Brown [58]
Native American knowledge of medicinal plants (pp. 66) (example of exchange of information between Native Americans and priests)	Boscana [63]
Junipero Serra’s leg treated by muleteer using local herbs (pp. 69) (example of exchange of information between Mestizo and priests) Friars unable to reduce death rate even with help from Native American shaman (pp. 156) (example of exchange of information between Native Americans and priests)	Castillo [59]
Dr. Prat searches for medicinal herbs (pp. 14) (example of the use of medicinal plant by Spanish explorers in California)	Engelhardt [64]
1812 survey of Missions asking about medicinal practices of Native Americans (example of exchange of information between Native Americans and priests)	Geiger and Meighan [23]
Gardens at Mission Delores (pp.58) (example of garden at a Mission where both medicinal plants from Europe and California were grown together for medicinal purposes)	Goerke [65]
Watercress reported at Mission San Gabriel (pp. 152) (example of medicinal plant native to both Spain and California observed at a Mission) Father Font identifies flora (pp. 176) (example of priest identifying native plants in California and referencing them to plant species in Spain of medicinal value) Anza becomes sick and is treated with medicinal (pp. 187) (example of exchange of information between Native American and Spanish explorers)	Guerrero [60]
Shared indigenous knowledge (pp. 33) (example of exchange of information between Native Americans and priests)	Kryder-Reid [66]
*Neophytes* were sometimes dispatched by the priests to collect medicinal plants from the wild (p. 576) (example of exchange of information between Native Americans and priests)	Engelhardt (1922)
At Mission San Jose the Native Americans retained their native customs (pp. 50-53) (example of Native Americans continuing their use of medicinal plants at the Missions)	Milliken [67]
Continued practice of native medicine at Soledad Mission (pp. 119) (example of Native Americans continuing their use of medicinal plants at the Missions)	Sandoz (2004)
Practice of herbal medicine (pp. 173) (example of Native Americans continuing their use of medicinal plants at the Missions) Use of *Datura toothache* (pp. 175-178) (example of Native Americans continuing their use of medicinal plants at the Missions) Use of horehound (pp. 180-181) (example of Native Americans continuing their use of medicinal plants at the Missions)	Timbrook [68]
Gardens at San Buenaventura (pp.86) (example of Native American medicinal plants being planted in Mission gardens)	Webb [61]
Exchange of information about medicinal plants (pp.160-161) (example of exchange of information between Native Americans and priests)	Weber [69]
**2. Mission gardens and apothecary shops**	
Shaman cultivated medicinal herbs (pp. 44) (example of Native American medicinal plants being planted in Mission gardens)	Blackburn and Anderson [62]
Seed imported from Mexico for Mission gardens (example of plants from a variety of sources being planted in Mission gardens)	Brown [58]
San Carlos Mission garden (pp. 186) (example of Native American medicinal plants being planted in Mission gardens)	Guerrero [60]
San Diego Mission gardens (pp. 36) (example of Native American medicinal plants being planted in Mission gardens)	Kryder-Reid [66]
Mission San Buenaventura gardens (pp. 294) (example of Native American medicinal plants being planted in Mission gardens)	Lamb [70]
San Luis Rey Mission gardens (pp. 96, 98) (example of Native American medicinal plants being planted in Mission gardens)	Tac [71]
Native American gardens (pp. 60) (example of Native American medicinal plants being planted in Mission gardens) Mission San Luis Rey gardens (pp. 76) (example of Native American medicinal plants being planted in Mission gardens)	Webb [61]
Domestication of native herbs (pp. 125) (example of Native American medicinal plants being planted in Mission gardens) Apothecary shops (pp. 129-13) (example of Native American medicinal plants being planted in Mission gardens) Native Americans encouraged to domesticate local plants (pp. 133) (example of Native American medicinal plants being planted in Mission gardens) Specialized gardens at different Missions (pp. 134) Apothecary shops in all Missions (pp. 160) (example of Native American medicinal plants being planted in Mission gardens)	Weber [69]

Table 5 summarizes references that suggest eight reasons for the impediments to the transfer of information. These are the following:

(1) A significant power imbalance existed between the priests and the Native Americans.

(2) Priests thought the Native Americans were savage heathens or children who knew nothing.

(3) Language barriers to communication.

(4) Reduction in the availability of medicinal herbs due to the elimination of Native American burning and the introduction of Spanish livestock.

(5) Knowledge of medicinal plants was a source of power and income for the Native American shamans who did not want to share it.

(6) Structural organization of the administration of Missions left little time for direct communication between priests and neophytes.

(7) Knowledge of herbal medicine was lost at the Missions by the neophyte’s children and grandchildren.

(8) Transportation limitations during the Mission period may have limited reciprocal shipments of medicinal plants between Spain and California.

**Table 5:** Limitations to the exchange of information on medicinal plants

1. A significant power imbalance existed between the priests and the Native Americans	
Comments	Source
The power of the priests was maintained by the presence of soldiers at the missions (p. 22) (example of imbalance of power between priests and Native Americans)	Webb [61]
Priests used corporal punishment to enforce their power (p. 113) (example of imbalance of power between priests and Native Americans)	Castillo [59]
Native Americans avoided a sharing of their knowledge of medicinal plants and healing practices by conducting healing activities at night out of sight of priests from fear of losing power to the priests (47-51; 71-80, 97-100, 119-120) (example of imbalance of power between priests and Native Americans)	Geiger and Meighan [23]
2. Priests thought the Native Americans were savage heathens or children and their pagan ways should be suppressed	
Comments	Source
Boscana’s view of the character of the Native American (pp. 52) (example of disrespect on the part of priests for Native American knowledge) Spanish attitude toward Native Americans (pp. 64) (example of disrespect on the part of priests for Native American knowledge) Fray Lausen’s poor view of Native Americans (pp. 93-94) (example of disrespect on the part of priests for Native American knowledge) Friars harangued Native Americans about their “savage” way of life (pp. 119) (example of disrespect on the part of priests for Native American knowledge)	Castillo [59]
Boscana referred to shamans as “diabolical imposters” (pp. 236) (example of disrespect on the part of priests for Native American knowledge) Shamans practiced quackery (pp. 237-238) (example of disrespect on the part of priests for Native American knowledge)	Engelhardt [64]
Fr. Boscana’s views of Native Americans (example of disrespect on the part of priests for Native American knowledge)	Hanke [72]
Fundamental duty of missionaries is to eradicate what is harmful in Native American customs (pp. 128-129) (example of disrespect on the part of priests for Native American knowledge)	Kryder-Reid [66]
Spanish hold native culture in contempt (p. 30) (example of disrespect on the part of priests for Native American knowledge)	Langsdorff [73]
Priest force Native Americans to alter their traditional practices (pp. 59) (example of disrespect on the part of priests for Native American knowledge) Shamans considered sorcerers and wizards by priests (pp. 109) (example of disrespect on the part of priests for Native American knowledge) Controlling and acculturating Native Americans (pp. 110) (example of disrespect on the part of priests for Native American knowledge)	Lightfoot [21]
Missionaries sought to make Native Americans ashamed of their traditional ways of life (pp. 223) Native rituals and beliefs identified as work of the Devil (pp. 225)	Milliken [74]
Priest have contempt for Native American’s abilities (p. 52) (example of disrespect on the part of priests for Native American knowledge)	Rawls [75]
Priests prohibit Native American from dancing at San Gabriel Mission (pp. 5) (example of disrespect on the part of priests for Native American knowledge) Fr. Boscana compares Native Americans to monkeys (pp. 21) (example of disrespect on the part of priests for Native American knowledge) “denaturalizing” of Native Americans (pp. 92) (example of disrespect on the part of priests for Native American knowledge) Shaman practiced sucking of objects from bodies of the afflicted (pp. 118) (example of disrespect on the part of priests for Native American knowledge) Tribal lore kept secret by Shaman (pp. 181-182) (example of disrespect on the part of priests for Native American knowledge)	Sandos [76]
Native Americans viewed as deceivers (pp. 481) (example of disrespect on the part of priests for Native American knowledge)	Shipek [77]
Native Americans viewed as devil worshipers (pp. 68) (example of disrespect on the part of priests for Native American knowledge)	Skowronek [78]
Challenge to indigenous medicinal practice (pp. 17) (example of disrespect on the part of priests for Native American knowledge)	Wilken-Robertson [32]
**3. Language barriers to communication**	
Original languages spoken by some neophytes usurped by other languages spoken by neophytes from different tribes (pp.51) (example of disrespect on the part of priests for Native American knowledge) Native American languages unworthy of study or preservation (pp. 51) (example of disrespect on the part of priests for Native American knowledge) Widespread lack of Spanish among neophytes (pp. 128a) (example of barrier to sharing of information due to different languages) No record that teachers were sent or that the friars established to teach Native Americans Spanish (pp. 128b) (example of barrier to sharing of information due to different languages) Policy of not teaching Native Americans to read or write Spanish (pp. 128-129) (example of barrier to sharing of information due to different languages) Missionaries did not learn native languages (pp. 140) (example of barrier to sharing of information due to different languages)	Castillo [59]
Perseverance and hard work required of the missionaries to learn Native American languages (pp. 177) (example of barrier to sharing of information due to different languages)	Guerrero [60]
Missionaries should make greater effort to learn Native American languages (pp. 39) (example of failure of priests to learn native languages)	Rawl (1984)
Language barriers (pp. 26 and 45) (example of barrier to sharing of information due to different languages) Variety of crude and barbarian languages among the Native Americans (pp. 46) (example of barrier to sharing of information due to different languages) Native Americans born in the Missions learned Spanish (pp. 47) (example of greater opportunity of second generation neophytes to exchange information on medicinal plants) Interpreters employed to neophytes since most padres did not learn the native languages (pp. 48a) (example of barrier to sharing of information due to different languages) Only those Native Americans born in the Mission all speak Castilian (pp. 48b) (example of greater opportunity of second generation neophytes to exchange information on medicinal plants) After 1840 Native Americans reported to speak Spanish (pp. 308) (example of greater opportunity of second generation neophytes to exchange information on medicinal plants)	Webb [61]
Great variety of Native American languages and dialects (pp. 15) (example of greater opportunity of second generation neophytes to exchange information on medicinal plants) Majority of the friars taught neophytes in Spanish, rather than in their native languages (pp. 124) (example of greater opportunity of second generation neophytes to exchange information on medicinal plants)	Weber [69]
**4. Reduction in the availability of medicinal herbs due to the elimination of Native American burning and the introduction of Spanish livestock.**	
Subsistence practices constrained at Missions (pp. 79) (example of Native American customs, including medicinal practices constrained at the Missions)	Lightfoot [21]
Plant management practices by Native Americans that would have been curtailed around the Missions (pp. 83) (example of Native American customs, including medicinal practices constrained at the Missions) Native American spiritual practices connected to plant harvesting curtailed by Missionaries (pp. 84) (example of Native American customs, including medicinal practices constrained at the Missions)	Lightfoot and Parrish [30]
Cessation of native fire management practices (pp. 27-28) (example of land management practices used by Native American to promote medicinal plants constrained at the Missions) Change of lifestyle resulted in a loss of interest in traditional commodities (pp. 222) (example of Native American customs, including medicinal practices constrained at the Missions)	Milliken [74]
Use of fire by Native Americans (pp. 12) (example of land management practices used by Native American to promote medicinal plants constrained at the Missions)	Timbrook [18]
Spanish soldiers destroy Native American field by grazing (pp. 48-49) (example of land management practices used by Native American to promote medicinal plants constrained at the Missions) Native American burning to produce more seeds (pp. 81) (example of Native American land management practices used to promote medicinal plants) Native American burning (pp. 117) (example of Native American land management practices used to promote medicinal plants) Crespi’s observation of Native American burning (pp. 121-122) (example of Native American land management practices used to promote medicinal plants) Evidence of Native American burning (pp. 124) (example of Native American land management practices used to promote medicinal plants) Governor Arrillaga bans Native American burning in 1793 (pp. 126-127a) (example of land management practices used by Native American to promote medicinal plants constrained at the Missions) Moncada’s 1774-1777 observations of Native American burning (pp. 126-127b) (example of Native American land management practices used to promote medicinal plants) Longinos’ observation of Native American burning (pp. 129) (example of Native American land management practices used to promote medicinal plants) Native American use of fire to influence plant growth (pp. 134) (example of Native American land management practices used to promote medicinal plants) Medicinal plants encouraged by Native American burning (pp. 145) (example of Native American land management practices used to promote medicinal plants)	Blackburn and Anderson [62]
Adoption of Native Americans to colonist’s land management practices (pp. 27) (example of land management practices used by Native American to promote medicinal plants constrained at the Missions) Spanish authorities prohibit Native Americans from burning (pp. 45) (example of land management practices used by Native American to promote medicinal plants constrained at the Missions)	Wilken-Robertson [32]
**5. Knowledge of medicinal plants was a source of power and income for the Native American shamans who did not want to share it**	
Structure of shamanism among California Native Americans (pp. 55-56) (example of Native American power structure effecting the use of medicinal plants)	Bean [79]
Secret knowledge (pp. 3) (example of Native American power structure effecting the use of medicinal plants)	Boscana [63]
Continued native practice of medicine (pp. 110) (example of Native American power structure effecting the use of medicinal plants) Native practices took place in neophyte quarters (pp. 112-113) (example of Native American power structure effecting the use of medicinal plants) Priests lament continued pagan practices of shamans at missions (pp. 183) (example of difficulty priest had in curtailing Native American customs)	Lightfoot [21]
Shaman’s skills required a “lifetime’ of experience (pp. 132-133) (example of Native American power structure effecting the use of medicinal plants)	Margolin [80]
Shaman’s methods of healing (pp. 27-28) (example of Native American power structure effecting the use of medicinal plants)	Milliken [74]
Shamans were skilled at the arts of healing (pp. 10) (example of Native American power structure effecting the use of medicinal plants)	Rawls [75]
Neophytes preserved much of their culture after baptism without the knowledge of the priests (pp. 94) (example of Native Americans attempting to preserve their knowledge and use of native plants for medicinal purposes)	Sandos [76]
Different kinds of shamans (pp. 142) (example of Native American power structure effecting the use of medicinal plants) Shamans secretive about their remedies (pp. 173) (example of Native Americans attempting to preserve their knowledge and use of native plants for medicinal purposes)	Timbrook [68]
6. Structural Organization of the administration of Missions left little time for direct communication between priest and *neophytes*	
*Alcaldes* appointed by priests (pp. 112) (example of priests using intermediaries in dealing with Native Americans)	Lightfoot [21]
Priest’s organization of *neophyte* community at the missions (pp. 9) (example of priests using intermediaries in dealing with Native Americans)	Sandos [76]
Number of Spanish/Mexican people at the mission compared to number of *neophytes* (pp. 488) (example of the large numbers of Native Americans at themission compared to priest)	Shipek [77]
**7. Knowledge of herbal medicine lost by the*neophyte’s*children and grandchildren**	
Traditional customs forgotten at the missions (pp. 192) (example of knowledge lost by second and third generation neophytes)	Castillo [59]
Undermining of traditional knowledge from one generation to the next at the missions (pp. 221) (example of knowledge lost by second and third generation neophytes) Gradual impoverishment of Native American lifestyle at the missions (pp. 222) (example of knowledge lost by second and third generation neophytes)	Milliken [74]
Previous ways changed the longer neophytes were at the missions (pp. 157) (example of knowledge lost by second and third generation neophytes) Neophytes lost touch with their culture quickly at the northern mission, but not so quickly at the southern missions (pp. 181-182) (example of knowledge lost by second and third generation neophytes)	Sandos [76]
Impact of mission system on indigenous medical knowledge (pp. 17) (example of knowledge lost by second and third generation neophytes) Impact of historical processes on ethnobotanical knowledge (pp. 15-16) (example of knowledge lost by second and third generation neophytes)	Wilken-Robertson [32]
**8. Limitations to transportation**	
Spanish restriction of exclusion and restriction of foreign trade with their possessions in the New World would have limited the transport of medicinal plants back to Spain (pp. 436-437) (example of constraints on the transportation of medicinal plants) Every year a transport ship arrived in San Diego, Santa Barbara, Monterey, and San Francisco with supplies for the Missions. Priests were required to pay for and to pay for any materials shipped back to Spain. The costs restricted shipment of medicinal herbs. (pp. 437) (example of constraints on the transportation of medicinal plants) In 1825 Governor Echeandia forbid the missionaries to trade with any vessel outside of the four Presidio ports. This required the expensive transport of materials on the backs of mules from Missions distant from the ports (pp. 224) (example of constraints on the transportation of medicinal plants)	Engelhardt [64]
After 1810 California was cut off from Spain and Mexico due to the civil war taking place in Mexico. This caused the missions to become more dependent on local landscapes for food and basic goods (pp. 67) (example of constraints on the transportation of medicinal plants)	Lightfoot [21]
Native Americans received inadequate medical care because of limited supplies of medicines (pp. 251-252) (example of constraints on the transportation of medicinal plants)	Langsdorff (1927)

### Post-Mission Period

The list of medicinal plants used both by Natives Americans and Californios indicates a much greater sharing of medicinal knowledge following the secularization of the Missions [19, 43]. The lists indicate 148 taxa were used to treat 288 ailments in 14 therapeutic groups (Fig. 2). Forty-four (30%) of these 148 taxa occur on the list of medicinal plants used by the Native American prior to the Mission period, forty-two taxa (28.4%) were in use during the Mission period.

## Discussion

The results of this study suggest limited sharing of information about medicinal plants occurred during the Mission Period. There are direct reports of the sharing of information such as the dispatching of *neophytes* to collect food plants and herbs during times of shortages [64]. Additionally, the priest at eight of the Missions responded to the 1812 survey that the local Native Americans used plants for medicinal purposes. One might assume that some of these plants would have been exported to Spain because of their medicinal value. However, none of the 15 species most commonly used by Native Americans occurs on the registry of plants introduced to Mediterranean area during the eighteenth and nineteenth centuries ([48]; Flora [49, 52, 53, 81]). Furthermore, none of these California species were reported to have been grown in present-day herb gardens in northern Spain [37, 38]. The exchange of information on medicinal plants is further supported by the presence of both European and Californian species in present-day Mission gardens and apothecary shops further supports the exchange of information.

Much more evidence was discovered in this study to suggest many possible factors contributed to constraining the sharing of information about medicinal plants. These factors and the sources of information about these factors are presented in Table 5. We elaborate on these factors as follows:

### A significant power imbalance existed between the priests and the Native Americans

The priests maintained significant power over the Native Americans at the missions. Their power was enforced by corporal punishment and confinement of the neophytes who did not work or who behaved badly in the eyes of the priests [61, 82]. This power imbalance resulted in the *neophytes* hiding some information concerning medicinal plants and shaman treating neophytes out of sight of the priests [21, 79]. Any acknowledgment of the value of Native American herbs by the priests would have been a way of giving power to the Native Americans.

### Priests thought the Native Americans were savage heathens or children who knew nothing

Many of the priests regarded the Native Americans as pagan savages whose customs needed to be suppressed. Interest in or communication about native medicinal plants would have been considered a way of endorsing native beliefs that the priests were dedicated to eliminating.

### Language barriers to communication

Language was also a barrier to communication between the priests and the Native Americans. Several quite distinct languages and dialects were spoken by Native Americans living along the California coast. Although the Mission priests were expected to learn the native languages and instruct the Native Americans in their native languages this was seldom the case [59]. The language barrier was limited not only to the difficulty and reluctance of the Mission priests to learn the native languages, but also to the first generation of Native Americans neophytes who learned only a minimum of Spanish. Spanish was acquired by Native Americans born at the Missions [61], but this and subsequent generations of Mission born Native Americans had less knowledge of native medicinal plants to share with the priests.

### Reduction in the availability of medicinal herbs due to the elimination of Native American burning and the introduction of Spanish livestock

The use of land for farming and livestock grazing along with the elimination of Native American burning of the landscape resulted in fewer medicinal plants in the vicinity of the Missions [30, 62, 74]. The resulting lack of access to native medicinal plants further interfered with the transfer on information.

### Knowledge of medicinal plants was a source of power and income for the Native American shamans who did not want to share it

The power and income Native American shamans received from their use of medicinal herbs were values that they would not have wanted to give up. The shamans continued their treatment of sick Native Americans at the Missions, but not in situations where they would be observed by the priests ([21, 74]; Timbrook 2000). Since the shaman’s knowledge of healing was acquired over many years and was not shared with the general population of Native Americans [80], one would not have expected they would be eager to share it with the priests.

### Structural organization of the administration of Missions left little time for direct communication between priests and neophytes

The Missions were initially organized to be administered by only two priests. They were assisted by a limited number of soldiers, cowboys, farmers, and craftsmen brought from Mexico [77]. Wives of some of these individuals were put in charge of the girl’s and unmarried women’s dormitories. Others worked as cooks. The priests selected *neophytes* to serve as *acaldes* and *enfermeros* in intermediate positions between the assistants brought from Mexico and the common *neophytes* [76]. The priests organized the work force of *neophytes* into four classes: first—skilled artisans; masons, carpenters, etc.; second—fishermen, stockmen, herdsmen, cowboys, tallow makers, hide cleaners, butchers; third—horticulturalists who tended mission gardens; fourth—laborers and field hands [76]. This administrative structure was necessary to manage the large numbers of *neophytes* at the Mission and to raise food [67]. The administrative structure limited one on one communication between the *neophytes* and the priests except in the catechism classes initially conducted by the priests. The priests had limited contact with the Native American women, some of whom were lower-level shamans possessing considerable knowledge of medicinal plants [79].

### Knowledge of herbal medicine was lost at the Missions by the neophyte’s children and grandchildren

An important impediment to the transfer of knowledge of herbal medicine was the loss of such knowledge by the initial generation of *neophyte’s* children and grandchildren [32, 59, 74]. The individuals who were born at the Missions had fewer contacts with native medicinal plants than Native Americans living away from the Missions. Sandos [76] suggests that previous customs changed the longer the *neophytes* were at the Missions.

### Transportation limitations during the Mission period may have limited reciprocal shipments of medicinal plants between Spain and California

Transportation from Spain to California and vice versa during the Mission period was limited. Most materials brought from Spain were shipped to ports on the east coast of Mexico, transported over land to Puerto Vallarta, and then shipped to ports in San Diego, Santa Barbara, Monterey, and San Francisco. Occasionally, ships from Europe would travel around the tip of South America to reach ports in California. Prior to the Mexican revolution, at least one ship would arrive annually with supplies for the Missions. During the Mexican War of Independence (1810-1821) shipments to California were for the most part halted [21, 69]. The Spanish priests did import European plants, including medicinal plants for gardens at the Missions [24]; however, observers at the time reported that the Native Americans received inadequate medical care mostly because of limited supplies of medicines [23, 73, 82]. As transportation was limited, especially during the conflict between Spain and Mexico there may have been little opportunity to ship medicinal plants back to Spain or to import them.

A greater exchange of information occurred during the post-Mission Period. The high number of plants used for medicinal purposes might be explained by the closer working relationships that occurred on the local ranches between the Native Americans and the Californios. Furthermore, the Californios had less incentives to “deculturalize” the Native Americans. Preparation of 46 of the herbal remedies reported by Garriga included ingredients (e.g., milk, whisky, castor oil) that were not available to the Native Americans in pre-Spanish times [19]. This suggests a sharing of information between the Californios and the Native Americans. We believe the greater sharing of information about the medicinal use of plants during the secularization and post-secularization period was due to (1) more one-to-one interactions between the Californios and the Native Americans, (2) many of the Californios were mestizos whose mothers or grandmothers were Native Americans, and (3) the lack of pressure on the part of the Californios to suppress Native American beliefs.

## Conclusions

We conclude from this study that there was a limited transfer of information on the medicinal use of plants between the Native American and Spanish priests during the Mission period. Many factors related to the obligations of the priests, their attitudes toward the Native Americans, language barriers, and cultural differences interfered with a more complete sharing of information. A primary factor in the lack of transfer of medicinal information between the Native American and the priest was the imbalance of power. This imbalance of power kept the Native Americans from sharing information. The fact that none of the 15 most commonly used California species were not transported to Spain for medicinal uses presents an interesting question: were these plants not considered of superior value to the plants in Spain for the treatment of illnesses or did the Native American not share their knowledge of these plants with the priests? The magnitude sharing of information about medicinal plants between the Native Americans and the Californios increased in the post-Mission Period. This increase was due to a greater contact between the Native Americans and the Californios and a different relationship that existed between the two groups. Important aspects of this relationship were increased one-on-one communication, mestizo background of the Californios, and the lack of responsibility on the part of the Californios to convert the Native Americans to christianity.

## Data Availability

All data generated or analyzed during this study are included in this published article.
